# TRF2 interaction with nuclear envelope is required for cell polarization and metastasis in triple negative breast cancer

**DOI:** 10.1038/s41419-025-07415-4

**Published:** 2025-03-30

**Authors:** Eleonora Petti, Serena Di Vito, Roberto Dinami, Manuela Porru, Stefano Marchesi, Jeroen Lohuis, Pasquale Zizza, Sara Iachettini, Erica Salvati, Carmen D’Angelo, Angela Rizzo, Carmen Maresca, Flora Ascione, Anna Di Benedetto, Simonetta Buglioni, Andrea Sacconi, Paola Ostano, Qingsen Li, Antonella Stoppacciaro, Carlo Leonetti, Jacco van Rheenen, Paolo Maiuri, Giorgio Scita, Annamaria Biroccio

**Affiliations:** 1 Translational Oncology Research Unit, IRCCS—Regina Elena National Cancer Institute, Rome, Italy; 2 Department of Ecological and Biological Sciences (DEB), University of Tuscia, Viterbo, Italy; 3 IFOM ETS—The AIRC Institute of Molecular Oncology, Milan, Italy; 4 Department of Oncology and Haemato-Oncology, University of Milan, Milan, Italy; 5 Division of Molecular Pathology, Netherlands Cancer Institute, Oncode Institute, Amsterdam, the Netherlands; 6 Institute of Molecular Biology and Pathology, National Research Council, Rome, Italy; 7 Department of Pathology, IRCCS—Regina Elena National Cancer Institute, Rome, Italy; 8 UOSD Clinical Trial Center, Biostatistics and Bioinformatics, IRCCS—Regina Elena National Cancer Institute, Rome, Italy; 9 Cancer Genomics Lab, Fondazione Edo ed Elvo Tempia, Biella, Italy; 10 Department of Clinical and Molecular Medicine, Sant’Andrea Hospital, Sapienza University of Rome, Rome, Italy; 11 Department of Molecular Medicine and Medical Biotechnology, Università degli Studi di Napoli “Federico II”, Naples, Italy

**Keywords:** Breast cancer, Metastasis

## Abstract

The Telomere Repeat-Binding factor 2 (TRF2) contributes to cancer progression by both telomere-dependent and independent mechanisms, including immune escape and angiogenesis. Here, we found that TRF2, through its Basic domain, directly interacts with Emerin forming a complex, including Lamin A/C, Lamin B1, SUN1, and SUN2. Importantly, TRF2 association with the inner nuclear membrane is functional to the proper establishment of cell polarity, finally promoting productive 1D and 3D migration in triple negative breast cancer cells (TNBC). In line with this, a spontaneous model of TNBC metastasis, combined with intravital imaging, allowed us to demonstrate that TRF2 promotes cell migration at the primary tumor site and is required for the early steps of the metastatic cascade. In human breast cancers, aberrantly elevated TRF2 expression positively correlates with cancer progression, metastasis, and poor prognosis, identifying TRF2 as a potential target for novel therapeutic strategies against TNBC.

## Introduction

Telomeres are nucleoprotein structures located at the physical ends of eukaryotic chromosomes. In human, they are composed of TTAGGG DNA tandem repeats bound by a multi-protein complex named Shelterin that prevents natural chromosome ends from being recognized as DNA breaks [1]. Among Shelterin components, Telomere Repeat Binding Factors 1 and 2 (TRF1 and TRF2) specifically bind to telomeric double stranded DNA, while Protection Of Telomeres 1 (POT1) associates with the single-stranded TTAGGG repeats at the telomeric 3’-overhang. The other three shelterin proteins, TIN2, TPP1, and RAP1, are indirectly associated to telomeric DNA through protein-protein interactions with TRF1 and/or TRF2 and/or POT1 [1].

TRF2, the central member of Shelterin complex, acts as a master regulator of telomere integrity by favoring the folding of the 3’ single-stranded G overhang into the T-loop and by suppressing ATM-mediated DNA damage response and non-homologous end joining repair pathway [1]. TRF2 is composed by four domains: the N-terminal Basic domain binds branched DNA in a sequence-independent manner; the Homodimerization (TRFH) and the HINGE domains are involved in protein-protein interactions, while the C-terminal Myb domain specifically binds telomeric double stranded DNA [1–3]. TRF2 is not frequently mutated in cancers, but it results up-regulated in a large panel of tumors [4–6], including breast cancer where accumulation of TRF2 has been reported to occur during transformation and progression ensuring telomere protection and indefinite lifespan maintenance [7, 8]. However, TRF2 contributes to tumorigenesis also through telomere-independent mechanisms, such as immune escape and angiogenesis [6, 9–11]. Till now, its extra-telomeric functions have been ascribed to its ability to bind to interstitial telomeric sequences (ITS) dispersed throughout the human genome, eventually regulating gene expression in cooperation with other chromatin remodeling factors [6, 10–12]. In addition to telomeres and ITS, TRF2 has been reported to be important for the stability of other heterochromatic regions, such as pericentromeres [13].

TRF2 has also been reported to interact with many different classes of proteins, including telomeric accessories factors (e.g., Apollo) [14, 15], components of DNA damage response/repair pathways (e.g., PARP1, BRCA1) [16, 17], chromatin factors [18], proteins involved in DNA replication (e.g., ORC, RTEL) [19–21] and components of the nuclear lamina [22, 23]. In particular, Lamin A/C and Lamin B1 have been included in the plethora of TRF2 interactors and their role has been investigated in the stability of ITS and telomeres, respectively [22–24].

The nuclear envelope (NE) has been traditionally viewed as a physical barrier to preserve genetic material in eucaryotic cells. It is composed by two individual lipid bilayers: the outer nuclear membrane (ONM), that faces the cytoplasm, and the inner nuclear membrane (INM), that faces the nucleoplasm, separated by a luminal space. The ONM and the INM are connected by the LINC (linker of the nucleoskeleton and cytoskeleton) complex formed by two classes of transmembrane proteins: KASH domain-containing proteins (Nesprins) at the ONM and SUN domain-containing proteins (SUN1, SUN2) at the INM which interact each other in the luminal space [25]. Connected with the cytoskeleton on one hand and with the nuclear lamina on the other, this complex is fundamental to mediate nucleus-cytoskeleton connections [26]. The nuclear lamina, a meshwork of lamins (A-type lamins and B-type lamins) and lamina-associated membrane proteins (i.e., LAP1, LAP2, Emerin, LBR) provides a scaffold to the NE and connects the INM to the chromatin [27]. A growing body of evidence reported that mutations or alterations of NE components such as Lamin A/C, Emerin and SUN1/2, cause nuclear structural changes, eventually leading to impaired nucleus-cytoskeleton connections and defects of nuclear positioning and cell polarity [28–32]. Moreover, alteration of nuclear mechanical properties of cancer cells, caused by mutations or alteration of NE proteins (e.g., Emerin), has been demonstrated to influence cell invasion ability, consequently impacting on metastatic dissemination in vivo [33, 34].

Here, we investigated the interplay between TRF2 and NE components and the functional consequences of this interaction in cancer. We found that TRF2, through its Basic domain, forms a complex with various NE components by directly interacting with Emerin, thus ensuring proper establishment of cell polarity and promoting directional cell migration in triple negative breast cancer (TNBC) models. Accordingly, loss of TRF2 strongly reduces spontaneous metastasis formation in vivo by perturbing local migration/invasion at the primary tumor site. We propose a novel mechanism by which TRF2, contributing to nucleus-cytoskeleton connections, directly promotes intracellular pathways leading to a more invasive phenotype.

## Results

### TRF2 forms a complex with inner nuclear membrane components by directly interacting with Emerin

**Fig. 1:**
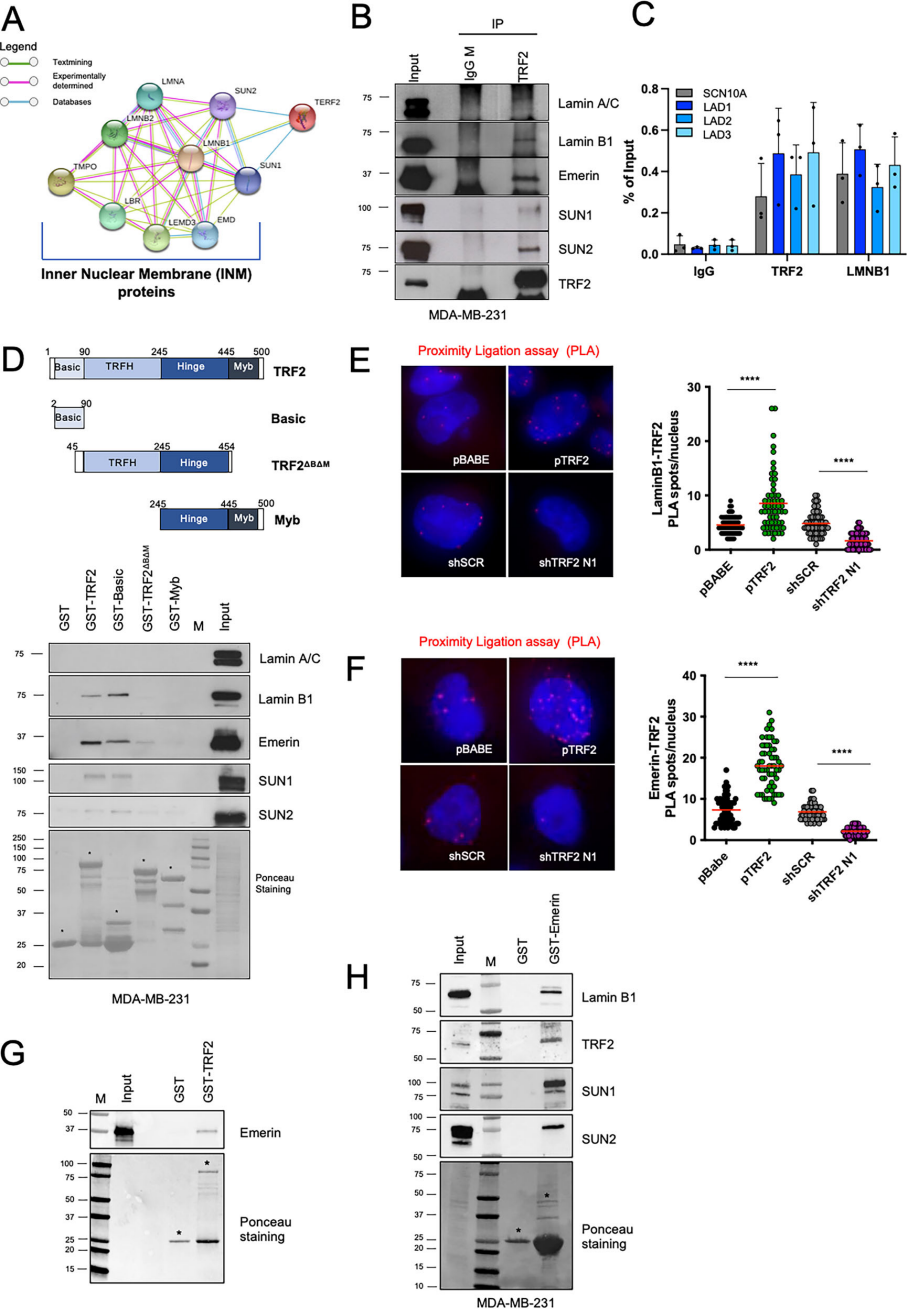
TRF2 forms a complex with INM proteins and directly interacts with Emerin. **A** Protein-protein interaction map (STRING) to explore TRF2-nuclear inner nuclear membrane proteins network. **B** Co-immunoprecipitation experiment with anti-TRF2 antibody in MDA-MB-231 cells. Mouse immunoglobulines (IgG M) were used as negative control. Immunoblots were probed with the indicated antibodies. **C** ChIP qPCR analysis shows the binding of TRF2 and Lamin B1 on the indicated LADs. SCN10A region, was used as positive control of LADs. **D** *Upper panel*, schematic representation of TRF2 and its deletion mutants used in this study. *Bottom panel*, the various GST-TRF2 proteins or GST alone were affinity-purified and incubated with lysates prepared from MDA-MB-231 cells, followed by Western blotting with indicated antibodies. **E** Proximity ligation assay to visualize interaction of TRF2 with Lamin B1 in MDA-MB-231 cells stable overexpressing or silenced for TRF2. *Left panel*: representative images. *Right panel*: quantification of TRF2-LaminB1 PLA spots per nucleus (*n* = 60 cells), red lines represent mean values. **F** Proximity ligation assay to visualize interaction of TRF2 with Emerin in MDA-MB-231 cells stable overexpressing or silenced for TRF2. *Left panel*: representative images. *Right panel*: quantification of TRF2-Emerin PLA spots per nucleus (*n* = 60 cells), red lines represent mean values. **G** In vitro GST pull-down assay with GST alone or GST-TRF2 in the presence of myc-Emerin recombinant protein. Following the pull-down, the samples were analyzed by western blotting using specific anti-Emerin antibody. **H** GST-Emerin protein or GST alone was affinity-purified and incubated with lysates prepared from MDA-MB-231 cells, followed by Western blotting with indicated antibodies. For (**D**) (*bottom panel*), (**G**, **H**), purified GST fusion proteins (indicated by the asterisks) were visualized by ponceau staining. For (**E**, **F**), Mann Whitney test was used to calculate statistical significance. *P < 0.05, **P < 0.01, ***P < 0.001, ****P < 0.0001.

Analysis of TRF2-INM proteins network, by STRING software, suggested a possible interaction of TRF2 with various components of the nuclear lamina and the INM (Fig. 1A). This hypothesis was experimentally validated by performing co-immunoprecipitation (with nuclear extracts) and chromatin-immunoprecipitation experiments in TNBC cells, showing that endogenous TRF2 forms a complex with Lamin A/C, Lamin B1, Emerin, SUN1 and SUN2 (Fig. 1B) and binds to some Lamina-Associated Domains (LADs) (Fig. 1C), heterochromatic regions which result from the association of peripheral chromatin with the nuclear lamina [35].

To identify the domain/s of TRF2 responsible for the interaction with the INM, we performed pull down experiments by using a recombinant TRF2 full length protein (TRF2 *wild type*, WT) or different fragments of TRF2 fused to GST, as illustrated in Fig. 1D, *upper panel*. In agreement with immunoprecipitation experiments, a large amount of endogenous Emerin and Lamin B1 was precipitated by GST-tagged TRF2 WT protein from nuclear extracts of MDA-MB-231 cells (Fig. 1D, *bottom panel*). On the contrary, SUN1 and SUN2 appeared faint, and Lamin A/C completely undetectable in recombinant GST-TRF2 eluate (Fig. 1D, *bottom panel*). Moreover, among the recombinant TRF2 fragments, only the GST-Basic domain retained the ability to precipitate the INM proteins (Fig. 1D, *bottom panel*). Consistently, deletion mutant of TRF2, lacking the Basic domain, was not enriched at LADs regions (Supplementary Fig. 1A, B).

Next, digging deeper into TRF2-Lamin B1 and TRF2-Emerin interactions, we found that, both Lamin B1 and Emerin appeared in close vicinity to TRF2 in interphase cells as specifically revealed by Proximity Ligation Assays (PLA) (Fig. 1E, F; Supplementary Fig. 1C–G). Interestingly, vitro-vitro pull-down experiments with recombinant proteins clearly demonstrated that only Emerin directly interacts with TRF2 (Fig.1G, Supplementary Fig. 1H). Consistently, GST-tagged Emerin was able to efficiently precipitate TRF2 and all the other components of the INM from MDA-MB-231 nuclear extract (Fig. 1H).

Collectively, these results revealed that TRF2, via its Basic domain, directly interacts with Emerin, thus forming a protein complex with various components of the INM.

### TRF2 interaction with the inner nuclear membrane contributes to the establishment of cell polarity

Mutations or alterations of nuclear lamins and other components of the NE can result in nuclear structural changes and nucleus-cytoskeleton uncoupling, eventually leading to altered nuclear positioning and improper establishment of cell polarity [30, 32, 36]. We therefore investigated whether TRF2, by participating to chromatin-cytoskeleton connections, might impact on nuclear structure as well as on cell polarization process.

**Fig. 2:**
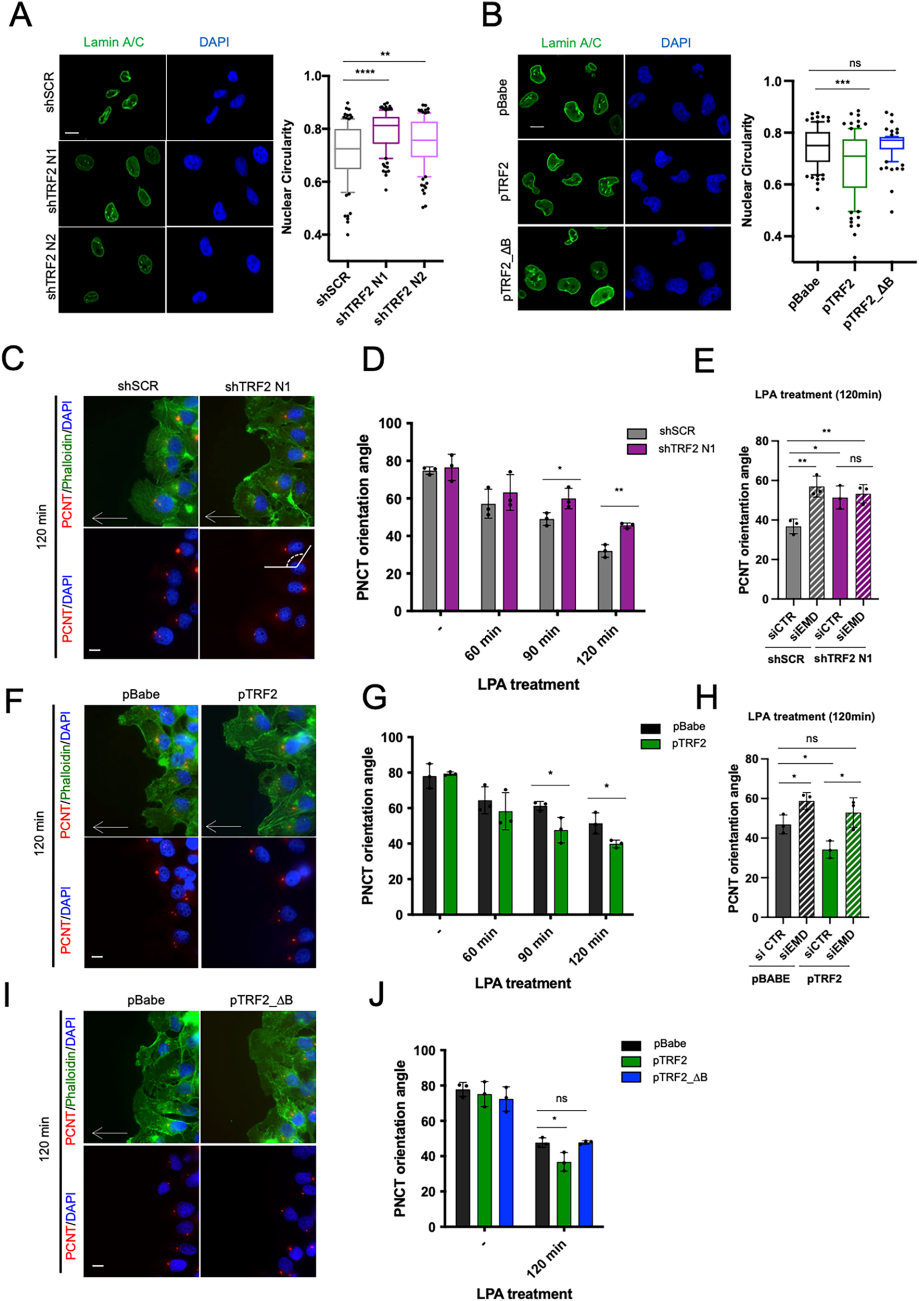
TRF2 association with Emerin and INM proteins is functional to the establishment of cell polarity. **A** *Left panel*: representative images of nuclear morphology of TRF2-depleted MDA-MB-231 cells visualized by immunofluorescence of Lamin A/C and DAPI (scale bar, 10 μm). *Right panel*: Box-and-whisker plots (10–90 percentile) of nuclear circularity index (*n* = 100). **B** *Left panel*: representative images of nuclear morphology of MDA-MB-231 cells stable overexpressing TRF2 WT (pTRF2) or TRF2_ΔB (pTRF2_ΔB) deletion mutant and their control (pBabe) visualized by immunofluorescence of Lamin A/C and DAPI (scale bar, 10 μm). *Right panel*: Box-and-whisker plots (10–90 percentile) of nuclear circularity index (*n* = 90). **C** Starved confluent monolayers of stable TRF2 interfered (shTRF2 N1) MDA-MB-436 cells and their respective control (shSCR) were scratch-wounded and stimulated by Lysophosphatidic acid (LPA) (10 μM). Cells fixed at different times upon LPA treatment, were stained with a centrosome marker (pericentrin), Phalloidin, and DAPI. Representative images at 120 min are shown. An example of measurement of pericentrin orientation angle is shown in shTRF2 representative image. For each cell, a first line was drawn from the center of the nucleus to the wound edge and a second line was drawn from the center of the nucleus to the center of the centrosome. The angle formed between the lines was measured. Scale bars, 10 μm. **D** Quantification of centrosome orientation angle in untreated or LPA treated cells for indicated times and experimental conditions. **E** Stable TRF2 interfered (shTRF2 N1) MDA-MB-436 cells and their respective control (shSCR) were transfected with indicated siRNA and processed as described in C. Quantification of centrosome orientation angle upon LPA treatment (120 min) in the indicated experimental conditions. **F** Stable TRF2 overexpressing MDA-MB-436 cells (pTRF2) and respective control cells (pBabe) were processed as described in C. Representative images at 120 min are shown. **G** Quantification of centrosome orientation angle in untreated or LPA treated cells for indicated times and experimental conditions. **H** Stable TRF2 overexpressing MDA-MB-436 cells (pTRF2) and respective control cells (pBabe) were transfected with indicated siRNA and processed as described in C. Quantification of centrosome orientation angle upon LPA treatment (120 min) in the indicated experimental conditions. **I** MDA-MB-436 stable overexpressing TRF2 WT (pTRF2) or TRF2_ΔB (pTRF2_ΔB) deletion mutant and their control (pBabe) were processed as described in C. Representative images at 120 min are shown. **H** Quantification of centrosome orientation angle in untreated or LPA treated cells for indicated experimental conditions at 120 min. For A, B, Mann Whitney test was used to calculate statistical significance. For (**D**, **E**, **G**, **H**, **J**), data are the mean ± SD (*n* = 3 independent experiments) and two-tailed *t* student test was used to calculate statistical significance. *P < 0.05, **P < 0.01, ***P < 0.001, ****P < 0.0001.

Lamin A/C nuclear staining revealed that both stable and transient knockdown of TRF2 induced a modified nuclear morphology characterized by an increased circularity index compared to control cells (Fig. 2A; Supplementary Fig. 2A). Conversely, overexpression of WT TRF2 (pTRF2), but not of deletion mutant lacking the Basic domain (pTRF2_ΔB), caused a reduction of nuclear circularity index (Fig. 2B). Then, we evaluated whether alterations of NE structure can impact the mechanical properties of the nucleus and of the entire cell. Atomic force microscopy (AFM) analysis revealed that TRF2 knockdown cells had a significantly reduced Young’s modulus, indicative of decreased stiffness, as compared to control (Supplementary Fig. 2B), suggesting that TRF2 expression alters nuclear and cell mechanics without significantly affecting expression or localization of major determinants of nuclear morphology and stiffness (Lamins, Emerin, LINC complex) (Supplementary Fig. 2C, D).

Next, with the aim to test a possible role of TRF2 on cell polarization, we took advantage of a starved confluent monolayer of another TNBC cell line (MDA-MB-436), stimulated to migrate by a scratch-wound followed by treatment with the serum factor lysophosphatidic acid (LPA) [37]. This agent triggers the synchronized polarization of previous starved cells. Importantly, by measuring pericentrin orientation angle at different time points after LPA treatment, we found that TRF2 knockdown induced a significant delay in cell polarization (Fig. 2C, D; Supplementary Fig. 2E, F). Interestingly, single silencing of Emerin caused a similar delay, while combined knockdown of TRF2/Emerin didn’t show an additive effect (Fig. 2E, Supplementary Fig. 2G). On the contrary, TRF2 overexpressing cells polarized more rapidly compared to their respective control (Fig. 2F, G; Supplementary Fig. 2E, H), and knockdown of Emerin abolished polarization advantage of TRF2 overexpressing cells (Fig. 2H, Supplementary Fig. 2I), strongly indicating the involvement of Emerin in the TRF2-mediated phenotype. Finally, we showed that the overexpression of TRF2_ΔB did not recapitulate the effects induced by TRF2 WT form on cell polarity (Fig. 2I, J; Supplementary Fig. 2J), demonstrating that TRF2 Basic domain is required for the establishment of cell polarity in TNBC cells.

### TRF2 is required for directional migration in TNBC cells

**Fig. 3:**
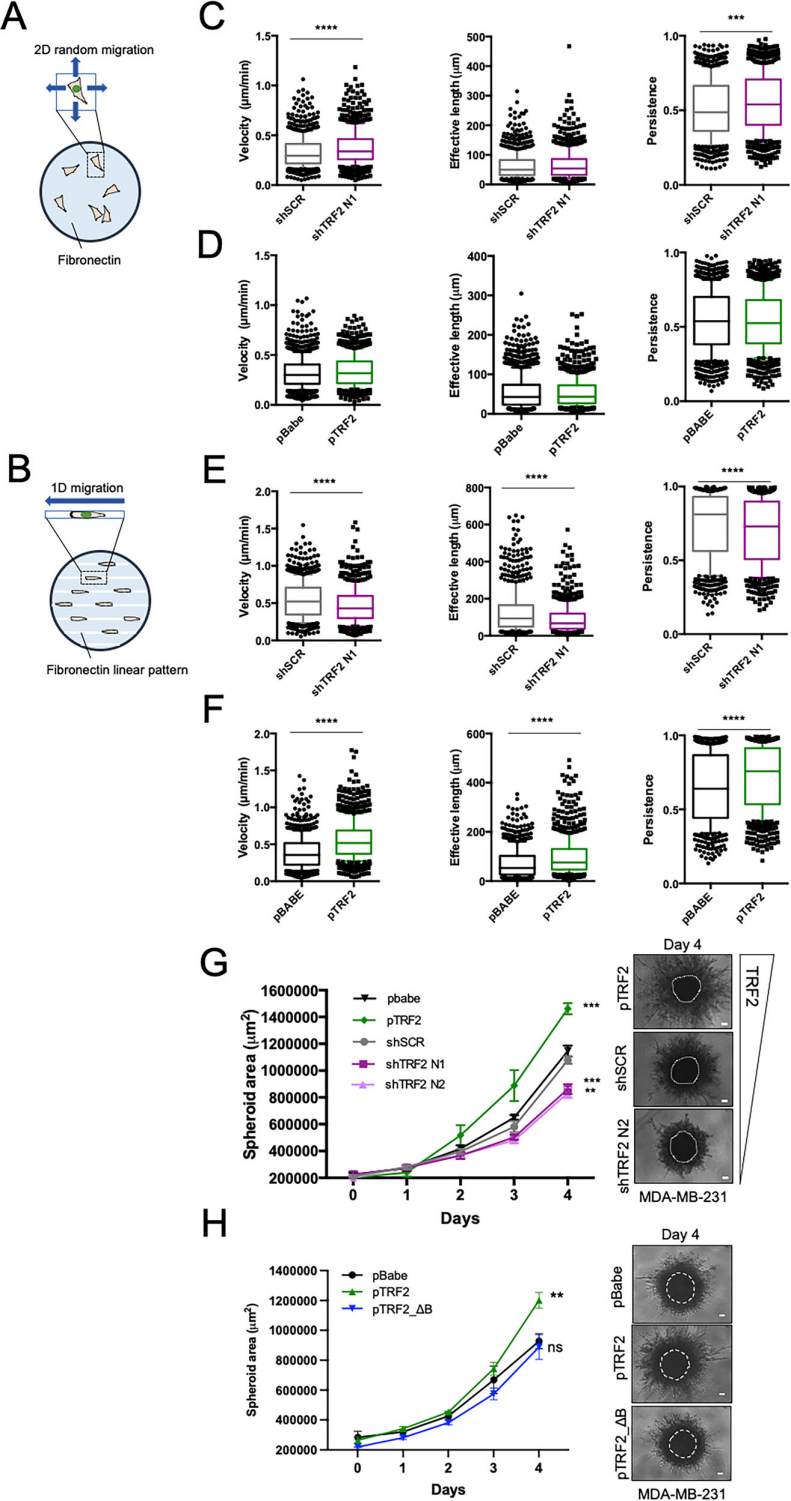
TRF2 is required for productive 1D and 3D migration. **A** Schematic representation of cells on fibronectin coated plates for random migration analysis (2D migration). **B** Schematic representation of cells on micropatterned lines, showing a polarized migratory morphology for 1D migration analysis. **C** Box-and-whisker plots (10-90 percentile) representing velocity, effective length, and persistence parameters of MDA-MB-231 cells stably depleted for TRF2 (shTRF2 N1) random migrating on the plate. **D** Box-and-whisker plots (10-90 percentile) representing velocity, effective length, and persistence parameters of MDA-MB-231 cells stably overexpressing TRF2 random migrating on the plate. **E** Box-and-whisker plots (10–90 percentile) representing velocity, effective length, and persistence parameters of MDA-MB-231 cells stably depleted for TRF2 (shTRF2 N1) migrating on micropatterned lines. **F** Box-and-whisker plots (10–90 percentile) representing velocity, effective length and persistence parameters of MDA-MB-231 cells stably overexpressing TRF2 migrating on micropatterned lines. **G** For 3D spheroid invasion assay, MDA-MB-231 stable cell lines were plated in a 96 well plate with a specialized ECM^R^ to drive spheroid formation of the cells. After 3 days (day 0) spheroids were embedded in Invasion Matrix^R^ and 3D invasion was visualized microscopically at indicated time points. *Left panel*: invasive activity into ECM was expressed as total spheroid area. Data are the mean ± SD (*n* = 3 independent experiments). *Right panel*: phase contrast images of representative spheroids for the indicated experimental conditions at day 4 are shown. Dashed circle indicates the size of spheroids at day 0. Scale bars, 100 μm. **H** MDA-MB-231 cells stable overexpressing TRF2 WT (pTRF2) or TRF2_ΔB (pTRF2_ΔB) deletion mutant and their control (pBabe) were subjected to 3D spheroid invasion assay. *Left panel*: invasive activity into ECM was expressed as total spheroid area. Data are the mean ± SD (*n* = 3 independent experiments). *Right panel*: phase contrast images of representative spheroids at day 4 are shown. Dashed circle indicates the size of spheroids at day 0. Scale bars, 100 μm. For (**C**, **D** and **E**, **F**), two independent experiments were performed. At least 600 cells were analyzed. *Kolmogorov-Smirnov* test was used to calculate statistical significance. For (**G**, **H**), Two-tailed *t* student test was used to calculate statistical significance. *P < 0.05, **P < 0.01, ***P < 0.001.

Establishment of cell polarity is a fundamental step in determining the direction of cell motion [38]. This prompted us to evaluate the impact of TRF2 on migratory behavior of TNBC cells by performing a battery of 2D and 1D single cell migration assays. We monitored cell migration by time-lapse microscopy in stably TRF2 depleted or overexpressing MDA-MB-231 cells that were seeded on uniformly fibronectin-coated plates (2D random migration) or fibronectin micropatterned lines (1D migration) (Fig. 3A–F). Automated cell tracking analysis showed that alterations of TRF2 level had no significant impact on the random locomotion of cells plated on flat surfaces (2D migration) (Fig. 3C, D); indeed, we only observed a slight increase in cell migration parameters in TRF2-depleted cells which was not paralleled by an opposite effect in TRF2 overexpressing cells (Fig. 3C, D). Accordingly, TRF2 expression had no significant impact on the organization of the actin cytoskeleton and focal adhesions (Supplementary Fig. 3A–H), nor it affected the ability of cells to adhere to fibronectin (Supplementary Fig. 3I, J).

In the 1D model system, where the cells are forced to polarize and move on linear patterns that mimic the ECM fibers of a constrained 3D environment [39], we found, instead, that TRF2 knockdown significantly reduced cell velocity, persistence, and effective traveled distance (Fig. 3E). Conversely, TRF2 overexpressing cells were significantly faster, more persistent and traveled a greater effective distance (Fig. 3F). Consistent with these results, in Boyden chamber Transwell assay, where the direction of migration is imposed by the serum gradient, we observed an increased number and a reduced number of migrated cells, respectively in TRF2 overexpressing and TRF2 depleted cells (Supplementary Fig. 4A).

In line with these results, 3D migration assays, where a tumor spheroid embedded in the extracellular matrix (ECM) invades isotropically in a multidirectional manner, revealed that TRF2 overexpressing spheroids displayed a significant increase of surface invasive area compared to their respective controls (Fig. 3G, Supplementary Fig. 4B). Conversely, silencing of TRF2 significantly impaired 3D spheroids invasion (Fig. 3G, Supplementary Fig. 4B). By comparing cell lines with the highest (pTRF2) and the lowest TRF2 levels (shTRF2 N1, shTRF2 N2), we observed a difference of about 50% in the spheroid surface area at day 4 in MDA-MB-231 and at day 6 in MDA-MB-436, respectively (Fig. 3G, Supplementary Fig. 4B). Moreover, a significant reduction of 3D spheroids invasion was also seen in MDA-MB-231 upon transient TRF2 knockdown by siRNAs or through the expression of an inducible TRF2 knockout system based on CRISPR-Cas9 technology [40] (Supplementary Fig. 4C, D), further confirming that TRF2 expression is required for effective 3D migration in the surrounding ECM.

Analyses of global/telomeric DNA damage response (DDR) revealed significant activation of DNA damage only upon transient silencing of TRF2 or inducible TRF2 knockout and not upon stable depletion of TRF2 in agreement with published data [10, 11, 41] (Supplementary Fig. 5A–G). Q-FISH analysis of cells seeded as in 2D or 1D migration as well as upon spheroid dissociation at the end of 3D invasion assay, demonstrated that stable depletion of TRF2 caused a slight increase of telomere length, independently from migration conditions (Supplementary Fig. 5H–M). Moreover, neither stable TRF2 overexpressing cells nor TRF2 depleted cells display significant changes in cell proliferation rate nor induction of apoptosis and/or senescence (Supplementary Fig. 6A–D), allowing us to exclude the contribution of proliferation to cell migration phenotype induced by TRF2 modulation.

Consistently with polarization experiments, migration analysis indicates that TRF2 is not essential for cell motility per se, but specifically influences the ability of TNBC cell to invade and move directionally. Moreover, we found that overexpression of TRF2_ ΔB had no impact on 3D migration of MDA-MB-231 spheroids (Fig. 3H), providing further evidence that the interaction of the Basic domain with the INM proteins mediates the pro-migratory effect of TRF2.

### TRF2 expression is required for metastasis formation in mouse models

The above results raise the interesting possibility that cell polarization and migration defects induced by TRF2 knockdown in vitro might affect cancer cell migration from primary tumor to distal sites, a multistep process known as metastasis.

**Fig. 4:**
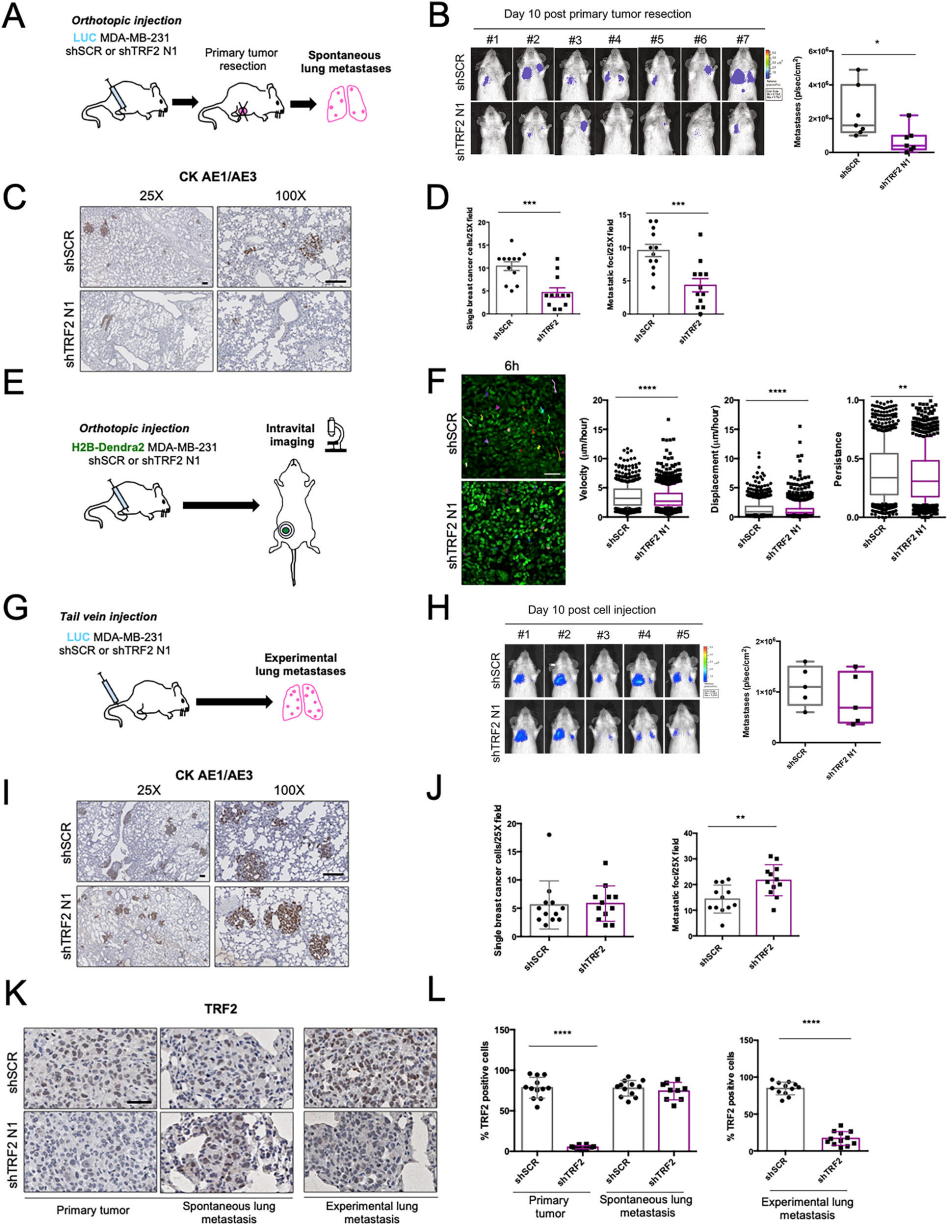
TRF2 knockdown reduces formation of spontaneous metastases in vivo. **A** Schematic representation of spontaneous metastasis experiment. Luminescent MDA-MB-231 shSCR and shTRF2 N1 were injected in the mammary gland of female NSG mice. Primary tumor was resected at day 19 and spontaneous lung metastases formation was monitored by bioluminescence imaging followed by ex vivo analyses. **B** *Left panel*, representative images of all mice ten days post primary tumor resection. *Right panel*, Box-and-whisker plot representing lung metastases bioluminescence intensity; shSCR (*N* = 7), shTRF2 (*N* = 7). Two-tailed *t* student test was used to calculate *P* value. **C** Representative images of Cytokeratin AE1/AE3 immunostained sections of lungs from spontaneous metastasis experiment. Scale bars, 100 μm. **D** Quantification of the number of single breast cancer cells and metastatic foci per 25X field. **E** Schematic representation of intravital experiment. MDA-MB-231 shSCR and shTRF2 N1 cells stably expressing H2B-Dendra2 were injected into the mammary gland of female NSG mice. After formation of primary tumors, animals have been subjected to intravital imaging. **F** *Left panel*, representative images of shSCR and shTRF2 N1 H2B-Dendra2 positive cells in their in vivo setting at the end point (6 h). *Right panel*, Box-and-whisker plots (10–90 percentile) of cell velocity, displacement/hour, and persistence; *n* = 900 cells from three mice per condition were analyzed. Two-tailed Mann Whitney test was used to calculate *P* value. **G** Schematic representation of experimental metastasis experiment. Luminescent MDA-MB-231 shSCR and shTRF2 N1 were injected in the tail vein of NSG mice and colonization of the lungs was monitored by bioluminescence imaging followed by ex vivo analyses. **H** *Left panel*, representative images of all mice ten days post cell injection. *Right panel*, Box-and-whisker plots representing lung metastases bioluminescence intensity; shSCR (*N* = 5), shTRF2 (*N* = 5). **I** Representative images of Citokeratin AE1/AE3 immunostained sections of lungs from experimental metastasis experiment. Scale bars, 100 μm. **J** Quantification of the number of single breast cancer cells and metastatic foci per 25X field. **K** Representative images of TRF2 immunostained sections of primary tumor, spontaneous and experimental lung metastases. Scale bars, 30 μm. **L** Quantification of the percentage of TRF2 positive cells in primary tumors and lung metastasis sections. For (**D**, **J**, **L**), twelve fields derived from three animals for condition were analyzed. Data are the mean ± SD, two-tailed *t* student test was used to calculate statistical significance. *P < 0.05, **P < 0.01, ***P < 0.001, ****P < 0.0001.

To investigate this possibility, we generated a spontaneous model of TNBC metastasis that reproduces the entire cascade from primary tumor formation to metastases appearance (Fig. 4A). Luminescent shSCR and shTRF2 MDA-MB-231 were orthotopically injected into the mammary fat pad of immune-compromised mice to allow primary tumor formation. Subsequently, primary tumors were resected, and the formation of spontaneous lung metastases was monitored by bioluminescence imaging (Fig. 4A). Under these experimental conditions, we did not observe any significant change in primary tumor growth as shown by quantitative bioluminescence imaging (Supplementary Fig. 7A, B). Immunohistochemistry (IHC) analysis of the xenografts revealed that primary tumors derived from TRF2 knockdown cells showed a slight reduction of ki67 positive cells, without any relevant change in DNA damage activation (γH2AX staining) and apoptosis induction (TUNEL assay), as compared to primary tumors derived from shSCR cells (Supplementary Fig. 7C, D). Quantitative bioluminescence imaging at day 10 post-resection, when all control mice showed detectable lung metastases signal, revealed that TRF2 knockdown drastically reduced spontaneous metastases formation (Fig. 4B). Notably, one mouse from shTRF2 group resulted completely negative for lung metastasis (Fig. 4B). For ethical reasons, mice were sacrificed at day 20, and lungs were subjected to ex vivo analyses. Hematoxylin and eosin staining (H&E) of lung sections revealed a significant reduction in the number and size of metastatic foci in mice injected with shTRF2 cells (Supplementary Fig. 7E). Consistently, by performing AE1/AE3 cytokeratin staining to detect the presence of metastatic deposits, we observed a reduction in the number of both single breast cancer cells and metastatic foci in the lungs of the mice injected with shTRF2 cells (Fig. 4C, D). Importantly, the reduction of spontaneous metastasis formation was confirmed after orthotopically injection of MDA-MB-436 TNBC cell line (Supplementary Fig. 8A–E). This result indicated that the pro-metastatic effect of TRF2 is a general phenomenon in TNBC.

We, then, used high-resolution time-lapse intravital imaging to assess whether cell migration defects at the primary tumor site may proceed the reduced metastasis. Our shSCR and shTRF2 MDA-MB-231 lines stably expressing a nuclear fluorescent protein (H2B-Dendra2) were injected into the mammary fat pad of NSG mice (Fig. 4E). Upon tumor formation, migration of tumor cells was imaged by surgically exposing the tumor and placing it on the imaging stage. Series of time-lapse z-stack images of the tumor were acquired every 30 min for at least 6 h (Fig. 4E). Cell tracking analysis revealed that, in line with our in vitro data (Fig. 3E), shTRF2 cells in the in vivo setting show reduced cell velocity, displacement and persistence compared to their control counterparts (Fig. 4F). These findings indicate that TRF2 knockdown impairs the local invasion of primary tumor cells in the surrounding ECM, thus delaying the early step of the metastatic process.

To exclude a possible involvement of TRF2 in the late phases of the metastatic cascade (extravasation and colonization of ectopic sites), we injected luminescent shSCR and shTRF2 MDA-MB-231 cells directly into the lateral tail vein of severely immune-compromised mice and monitored lung colonization by quantitative bioluminescence imaging (Fig. 4G). Under these experimental conditions, we observe no difference in lung metastasis formation in mice injected with shTRF2 cells compared to control animals (Fig. 4H), as confirmed by H&E analysis of the number and area of metastases and by AE1/AE3 cytokeratin staining on ex vivo lung sections (Fig. 4I, J; Supplementary Fig. 7F).

Finally, we measured TRF2 levels by IHC analysis on primary tumors and lung metastases from both orthotopic and intravascular injection models. As expected primary tumors derived from TRF2 knockdown cells maintained reduced TRF2 levels (Fig. 4K, L). Surprisingly, we found that spontaneous metastases derived from shTRF2 primary tumors expressed high levels of TRF2 as those derived from primary control tumors (Fig. 4K, L). On the contrary, TRF2 silencing was maintained in lung metastases resulting from colonization of intra-vein injected shTRF2 cells (Fig. 4K, L). Altogether these data demonstrate that TRF2 expression is required for the early steps of the metastatic cascade in TNBC.

### TRF2 expression correlates with breast cancer progression and is required for metastasis formation in human patients

**Fig. 5:**
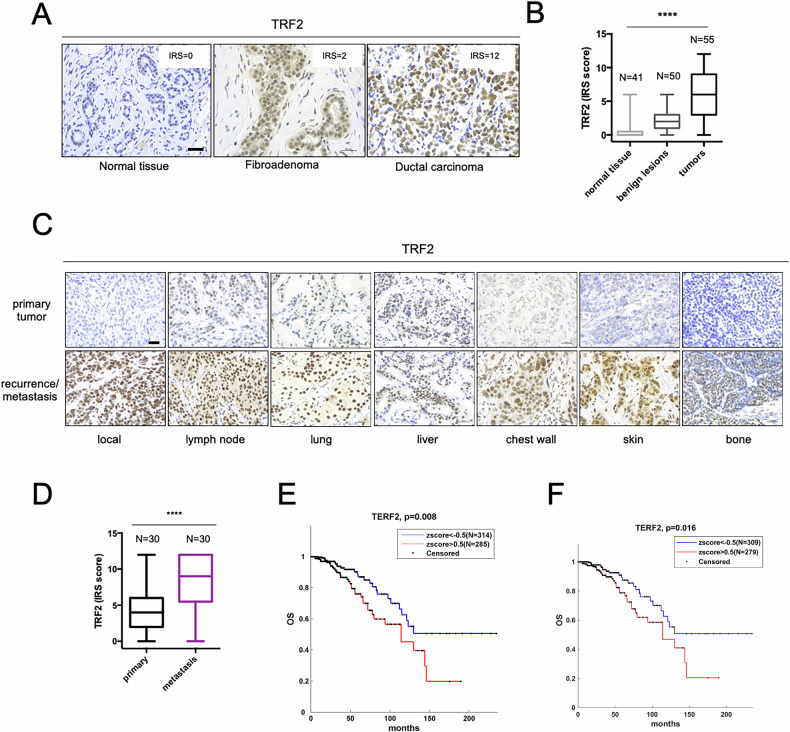
TRF2 expression positively correlates with breast cancer progression and metastasis. **A** Representative images of immunohistochemistry evaluation of TRF2 expression in Normal breast tissue, Fibroadenoma (benign lesion), and Ductal carcinoma (malign tumor). Scale bars, 30 μm. **B** Quantification of TRF2 expression (Immunoreactive score) in Normal breast tissue (*N* = 41), benign lesions (*N* = 50), and malign tumors (*N* = 55) surgically treated in IRCCS-Regina Elena National Cancer Institute. Statistical significance was calculated by one-way ANOVA (****P < 0.0001). **C** Representative images of immunohistochemistry evaluation of TRF2 expression in primary TNBC and their matched metastatic lesions (local recurrence and different distant organs). Scale bars, 30 μm. **D** Quantification of TRF2 expression (Immunoreactive score) in primary TNBC and their autologue metastatic lesions from patients surgically treated in IRCCS-Regina Elena National Cancer Institute (*N* = 30). Statistical significance was calculated by Wilcoxon matched-pairs rank test (****P < 0.0001). Overall survival (OS) evaluated by Kaplan-Meier curves on all stages (**E**) or stages I-II-III (**F**) BC patients from the TCGA dataset. Patients were stratified on the basis of TRF2 mRNA expression. Statistical significance was calculated by logrank test (*P < 0.05).

To test the clinical relevance of our findings, we investigated the correlation between TRF2 expression and breast cancer formation and progression in human patients. Human samples of normal breast tissue (*N* = 41), benign lesions (*N* = 51) or malignant tumors (*N* = 55) surgically treated at Regina Elena National Cancer Institute were analyzed for TRF2 protein expression by IHC analysis (Fig. 5A, B). Interestingly, TRF2 Immunoreactive score (IRS), obtained by multiplying the TRF2 positive cells proportion score for the TRF2 staining intensity score, progressively increased from normal tissue to benign lesions and became even higher in malignant tumors (Fig. 5A, B). To establish whether TRF2 expression varied among the different subtypes of malignant tumors, we used a larger cohort of breast cancer patients (*N* = 1026) from The Cancer Genome Atlas (TCGA) dataset. TRF2 mRNA expression progressively increased with the increasing aggressiveness of tumor subtypes, reaching the highest level in the basal subtype (Supplementary Fig. 9A). According to this, TRF2 expression was higher in breast tumors negative for estrogen, progesterone, and HER2 receptors (Triple Negative), characterized by the most aggressive clinical course, early relapse and poor outcome [42] (Supplementary Fig. 9B). Moreover, TRF2 expression is higher in stage II-III-IV as compared to stage I breast cancer patients (Supplementary Fig. 9C).

To address whether TRF2 expression is important for breast cancer metastasis in human patients, we performed an analysis of TRF2 expression on primary TNBC and their matched metastatic lesions—from a cohort of patients surgically treated in our institute (*N* = 30). We found a significant increase of TRF2 expression in metastatic lesions compared to primary tumors regardless the site of metastasis (local recurrence or different distant organs) and independently from the route of spread (blood or lymphatic system) (Fig. 5C, D). These results indicate that TRF2 plays a critical role in promoting metastatic process in human TNBC patients. Finally, by stratifying patients from TCGA dataset on the basis of the TRF2 mRNA expression (TRF2 low 314/599; TRF2 high 285/599), we found that high levels of TRF2 correlate with a worse clinical outcome of BC patients also by excluding stage IV patients (Fig. 5E, F).

Altogether, these data support the concept that TRF2 expression positively correlates with breast cancer aggressiveness, metastasis formation, and poor clinical outcome of breast cancer patients.

## Discussion

In this work, we found that, in addition to the recently demonstrated interaction with Lamin A/C and B1 [22–24], TRF2 also binds to other, previously unreported, NE proteins such as Emerin and two members of the LINC complex (SUN1 and SUN2), strengthening the existence of a close association of TRF2 with the INM. The interplay between TRF2 and the NE was further supported by new evidence demonstrating the presence of TRF2 on chromatin regions located at the nuclear periphery known as Lamina associated domains (LADs). This is in line with the idea that, in addition to telomeres, TRF2 is important to maintain the stability of other heterochromatic regions in the genome [13]. Moreover, we provided new insights by identifying the Basic domain as the main region of TRF2 responsible for the association with NE proteins and LADs. TRF2 Basic domain is reported to bind branched DNA structures [43–46] and to mediate protein-protein interactions such as those with core histones (H3/H4, H2A/H2AB), ORC1, DNA pol β and FEN1 [18, 47, 48], suggesting that this domain could be involved in both NE proteins interaction and binding to LADs DNA.

Digging deeper into the nature of protein-protein interactions, we identified Emerin as the key factor of the complex able to directly bind TRF2. In addition to these new insights into TRF2-NE association, our work attributed a novel function to this interaction impacting on the maintenance of nuclear mechanics and directional cell migration and metastasis, a hallmark of highly aggressive TNBC cells.

Specifically, we found that TRF2 alters nuclear shape and cell stiffness, features affecting migration/invasion capability of cancer cells [49, 50], without significant changes in the expression or localization of main NE components.

Moreover, we demonstrated that TRF2 expression impacts on centrosome orientation during cell polarization process. In line with nuclear positioning and cell polarization defects observed in laminopathies or upon alteration of Lamin A, Emerin, or LINC complex in fibroblasts or myoblasts [51], we also demonstrated that, similarly to TRF2 knockdown, also silencing of Emerin, direct TRF2-interactor, induced polarization defects in our TNBC model and that combined knockdown of TRF2/Emerin didn’t show an additive effect, suggesting that the two proteins are acting on the same axis. Moreover, knockdown of Emerin in TRF2 overexpressing cells rescued polarization advantage, further confirming the involvement of Emerin in the TRF2-mediated phenotype. Consistently, overexpression of deletion mutant of TRF2 lacking the Basic domain did not recapitulate effects induced by TRF2 WT overexpression on cell polarization, indicating that TRF2 Basic domain, mediating the interaction with Emerin and the other nuclear membrane components, is required for the establishment of cell polarity in TNBC cells. Based on these data TRF2 becomes part of the mechano-transduction machinery of the cells, participating to the communication between the cytoskeleton and nuclear interior.

In agreement with cell polarity defects, single cell migration assays upon modulation of TRF2 expression revealed that lack of TRF2 does not impair cell motility “*per se”* but prevents TNBC cells from performing directional and productive linear migration. Indeed, we found a significant effect of TRF2 on 1D and 3D migration, where the cells are forced to polarize and directionally migrate in a constrained environment, in a manner that appeared to be independent from telomere length and DNA damage activation. Importantly, also in 3D migration assay, overexpression of deletion mutant of TRF2 lacking the Basic domain did not recapitulate effects induced by TRF2 WT overexpression, indicating that TRF2 interaction with the INM is required to drive the pro-migratory phenotype. Our data are in line with previous studies which reported a role of TRF2 on cell migration in endothelial cells and renal cell carcinoma cells [5, 52] without providing a deeper characterization of the migratory behavior induced by overexpression/depletion of TRF2.

The above results raise the interesting possibility that TRF2 may impact on cancer metastasis. Multiple in vivo experimental approaches allowed us to demonstrate that TRF2 affects the initial local invasion of cancer cells at the primary tumor site, one of the key steps of the metastatic cascade. Indeed, TRF2 knockdown significantly reduced the appearance of spontaneous metastases induced by orthotopic injection of TNBC cells while no effect was observed by direct intra-vein injection of the same cells excluding a possible role of TRF2 in the extravasation and colonization steps. Importantly, high-resolution time lapse intravital imaging by monitoring single cell migration exactly at the primary tumor site allowed us to confirm that impairment of the metastatic process is directly linked to reduced velocity, displacement, and persistence of TRF2 depleted cells in their in vivo setting. These experiments, together with 3D spheroid migration assay, by covering long periods of time, allowed us to consider the reduction of cell migration, induced by stable TRF2 knockdown, as a persistent phenomenon.

Finally, we addressed clinical relevance of our findings by identifying a positive correlation between TRF2 expression and breast cancer progression in human patients by integrating analyses from TCGA dataset and from a cohort of patients surgically treated at Regina Elena National Cancer Institute. We found that TRF2 protein expression increases during the progression from normal tissue to benign lesion up to malignant lesion. Moreover, TRF2 mRNA level is higher in TNBCs and positively correlates with stage progression and a worse clinical outcome in BC patients (TCGA). In addition, our data in mouse models and human patients showed that TRF2 is overexpressed in the metastatic lesions, compared to the primary tumors, regardless the dissemination site, identifying TRF2 as a marker of metastasis and prognosis in TNBC.

**Fig. 6:**
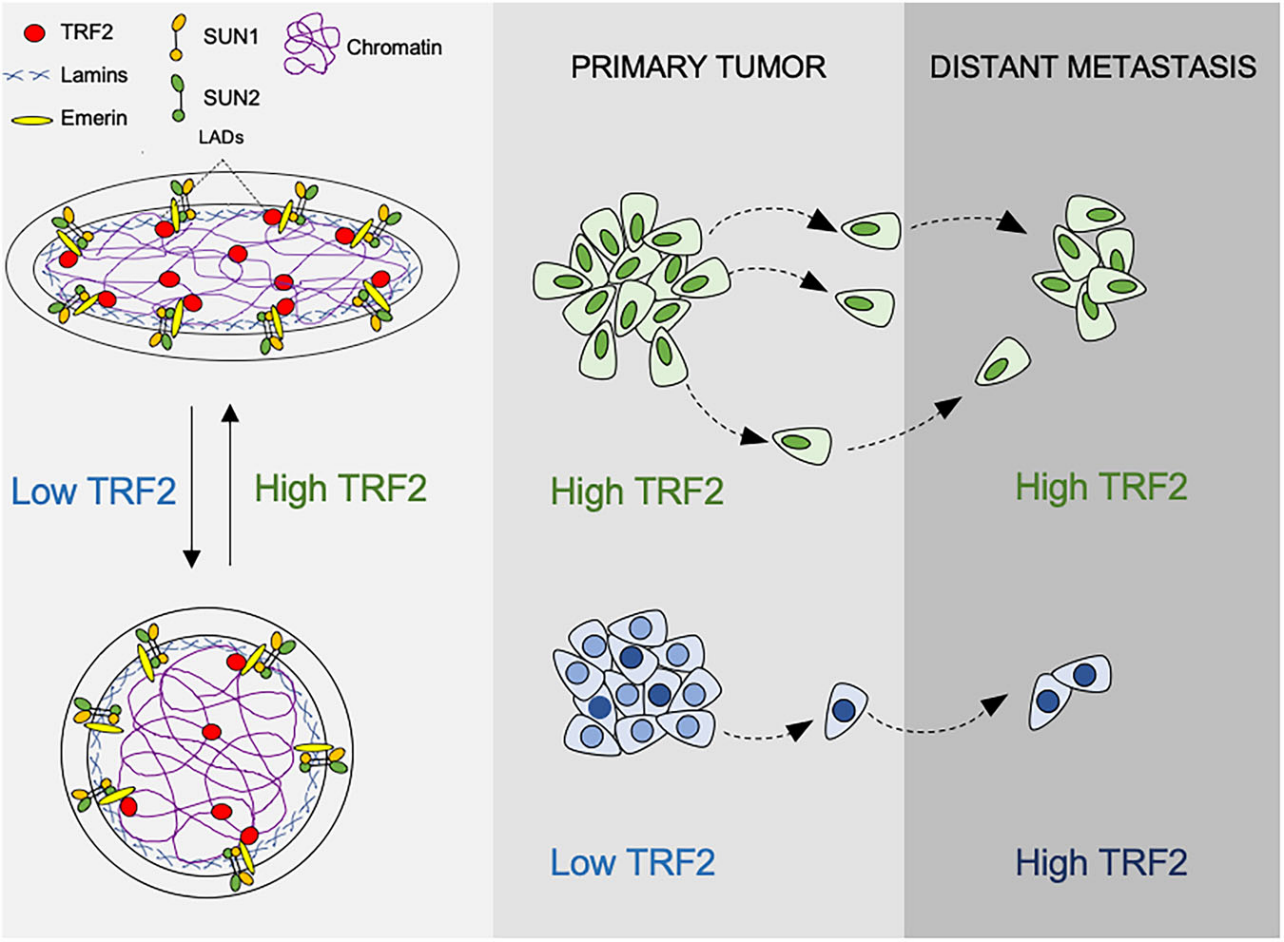
The association of TRF2 with the inner nuclear membrane complex occurs through a direct interaction with Emerin. This allows proper establishment of cell polarity and directional migration of cancer cells from primary tumor to distant site of the body giving rise to spontaneous metastasis. Knockdown of TRF2 increases nuclear circularity, reduces cell stiffness, impairs cell polarity and directional cell migration, finally leading to reduced metastasis formation. TRF2 expression results higher in the metastatic lesion as compared to matched primary tumor.

Collectively, our data demonstrated that TRF2, along with the regulation of cell-extrinsic pathways (tumor immunosurveillance and angiogenesis) [6, 10, 11], has a direct effect on cancer cell intrinsic features such as directional cell migration and metastasis (Fig. 6). These molecular and pre-clinical results strongly support the use of the recent miRNA-based anti-cancer approach against TRF2 for treatment of TNBC patients [53].

## Materials and methods

### Cell lines, culture conditions, transfections, and infections

Human TNBC cell lines MDA-MB-231, MDA-MB-436, and 4T1 mouse breast cancer cells were purchased from American Type Culture Collection (ATCC). All the cell lines were grown in high glucose Dulbecco modified eagle medium (DMEM; Invitrogen, Carlsbad, CA, USA) supplemented with L-glutamine, Penicillin/streptomycin and 10% fetal bovine serum (FBS, Hyclone) and routinely tested for mycoplasma contamination. Stable cell lines were generated as previously described [11]. Briefly, TRF2 overexpressing cells (pBabe-puro-mycTRF2, pBabe-puro-mycTRF2ΔB) and the control counterpart (pBabe-puro-Empty) [54] were obtained by infecting the cells with amphotropic retroviruses generated into Phoenix packaging cells transfected with retroviral vectors, using the JetPEI reagent (Polyplus, New York, NY, USA), according to the manufacturer’s instructions.

For stable silencing of TRF2 gene, cells were infected with lentiviral particles produced into HEK293T cells transfected with the packaging pCMVR8.74 and the envelope pMD2.G vectors in combination with the vectors encoding either for a scramble short hairpin sequence (shSCR; N2040 targeting *Escherichia coli* DNA polymerase) or for one of the two short hairpin sequences directed against TRF2 (shTRF2_N1: N2571 TRCN0000004811 or shTRF2_N2: N2573 TRCN0000004813 which were a gift from Prof. Stefan Schoeftner, University of Trieste). Early passages of stably infected cells were used for all experiments.

For in vitro single cell migration analysis, MDA-MB-231 stably TRF2 overexpressing /depleted cell lines were transduced with H2B-GFP retroviral vector (pBABE-H2BGFP) for nuclear staining and positive cells were selected by FACS sorting to reach a homogeneous cell population. For transient RNA interference experiments, siTRF2 (TRF2 Modified siRNA GEHCI-000246 GCAGAAGUGGACUGUAGAAUU, Dharmacon Inc., Chicago, USA) and siCTR (Control siRNA-A4, sc-37007, Santa Cruz Biotechnology; CA, USA Lot D1421) were transfected into cells with Interferin (Polyplus) according to the manufacturer’s instructions. For transient Emerin knockdown, a pull of three different siRNAs (Origene, SR301395A: rUrArGrArGrUrArArArGrCrGrUrCrCrUrCrUrUrUrCrUrUrGrGrGrA; SR301395B rUrCrUrArGrUrCrGrArArUrUrCrArArGrUrCrArGrArGrArArGrCrU; SR301395C: rGrUrCrGrArArUrUrCrArArGrUrCrArGrArGrArArGrCrUrArUrArA) or a Negative Control siRNA (SR300004, Origene) was transfected with Lipofectamin RNAiMAX (Thermo Scientific) according to the manufacturer’s instructions.

Generation of inducible CRISPR KO MDA-MB-231 cell line was performed as described in Kim et al. [40] thanks to vectors kindly provided by Zhou Songyang. Briefly, MDA-MB-231 cells stably expressing doxycycline-inducible Cas9 were generated by lentiviral transduction. A single clone with efficient Cas9 induction was selected. Vectors expressing dual shRNAs were introduced in the Cas9-inducible clone by lentiviral transduction followed by selection with specific antibiotics. For Induction of Cas9 and efficient knockout of TRF2, 5 days of incubation in 1 μg ml^−1^ of doxycycline was used.

For intravital imaging analysis, MDA-MB-231 stably depleted for TRF2 and expressing H2B-Dendra2 were generated by transfection with a specific vector (pMiniTol-EF1a-H2B-Dendra2) in combination with trasposase vector (pCS3-Trasposase), followed by FACS sorting.

For generation of spontaneous and IV metastasis models, MDA-MB-231 stably depleted for TRF2 were infected with lentiviral luciferase vector pRRLSIN.cPPT.RFPL4b (Addgene) that allows stable expression of luciferase for in vivo monitoring of metastases.

### Protein extracts and western blotting

Cell lysates were prepared using a Lysis buffer (50 mmol/L Tris-HCl (pH 7.5) 250 mmol/L NaCl, 5 mmol/L EDTA, 0,1% Triton) and western blotting was performed as previously described [55]. Primary antibodies are listed in Supplementary Table 1. For Uncropped western blot images see Original Data File.

### Immunoprecipitation (IP)

Nuclear cell extracts were obtained by a sequential lysis with buffer A (10 mM Hepes pH 7.9, 10 mM KCl, 0.1 mM EDTA, 0.1 mM EGTA, 0.6% NP-40, 1 mM DTT and 1 mM PMSF) and buffer C (20 mM Hepes pH 7.9, 400 mM NaCl, 0.1 mM EDTA, 0.1 mM EGTA, 1 mM DTT and 1 mM PMSF), which resulted respectively in cytosolic and nuclear fraction isolation. Protein concentration was determined and 500 μg of nuclear fraction were used for each IP. Magnetic beads A and G (Dynabeads, Thermo Fisher Scientific) were washed with PBS 0.02% Tween-20 (Wash Buffer) and bound to 4 μg of specific antibody or mouse IgG as negative control (15 min of incubation at room temperature). Then antibody-beads complexes were washed and incubated with nuclear extracts for 45 min at room temperature. Immunoprecipitates were washed, eluted from the magnetic beads, and boiled at 95 °C for 5 min before SDS page.

### Purification of GST fusion proteins

All tested GST-tagged proteins were expressed in Escherichia coli as reported in [56]. Bacteria expression vectors pGEX2T-GST, -TRF2wt, -TRF2basic, -TRF2ΔBΔM and -TRF2myb were kindly provided by Paul M. Lieberman’s lab [47], while Emerin-GST were kindly provided by Bulmaro Cisneros lab [57].

### GST pull-down assays

Vitro-Vitro *pulldown assay*. 5 pmol of GST-TRF2 recombinant protein were incubated with 5 pmol of myc-Emerin or myc-LMNB1 recombinant protein (Origene, TP300643, and TP301604, respectively) in agitation in 1 ml of GST incubation buffer (20 mM Tris–HCl pH 8, 100 mM KCl, 1 mM EDTA and 0.2% Triton) over night (ON) at 4 °C. Successively, in order to precipitate GST recombinant proteins, 60 μl of Glutathione Sepharose 4B matrix (GE Healthcare) were added to the buffer and incubated in agitation at RT for 2 h. After five washes in GST incubation buffer, the precipitated proteins were eluted from Glutathione Sepharose 4B matrix (by adding reducing protein loading buffer and incubating the samples at 95 °C for 5 min) and successively run in a denaturating SDS page.

Vitro-Vivo *pull-down assay*. Nuclear protein fractions were obtained from MDA-MB-231 cells lysed as described in [56]. After protein concentration determination with Pierce™ BCA Protein Assay Kit (Thermo Fisher Scientific), 500 μg of nuclear protein were incubated ON at 4 °C with 60 μl of the recombinant protein-conjugated resin in 1 ml of a buffer containing 50 mM Tris–HCl pH 7.5, 150 mM NaCl, 5 mM EDTA, 0.1% NP-40, 1 mM DTT, and 1 mM PMSF. After five washes with a buffer containing 50 mM Tris–HCl pH8, 200 mM NaCl, 0.25% NP-40, and 0.5 mM PMSF, beads were resuspended in 40 μl of reducing protein loading buffer and incubated at 95 °C for 5 min. Supernatants were run on a denaturating SDS page.

### Proximity Ligation Assay (PLA)

Cells were seeded on gelatin coated slides. After 24 h cells were fixed and permeabilized as described for Immunofluorescence, then they were processed according to protocol of Duolink® PLA Fluorescence (Sigma Aldrich). Images were acquired with Leica DMIRE deconvolution microscope equipped with a Leica DFC 350FX camera and elaborated by a Leica LAS X software (Leica, Solms, Germany). Number of the spots per cell were counted by visual inspection on the maximum projection resulting from multiple z-stack images.

### Chromatin immunoprecipitation (ChIP)

ChIP assay was performed as reported in [12]. Briefly, for each condition 7 × 10^5^ MDA-MB-231 cells were seeded and, 3 days after, were fixed with 1% of formaldehyde. Whole cells (6 × 10^6^) were firstly lysed in 1,2 ml of lysis buffer and disrupted by using a Dounce homogenizer with B pestle (tight) 5 times. Next, the nuclei were lysed in 350 μl of SDS lysis Buffer (Tris-HCl pH 8 1 M, EDTA 0,5 M, SDS 20%) and sonicated 3 times for 5 min setting high level by using the biorupter standard (Diagenode Inc., NXT-Dx Belgium). The average length of sonicated chromatin considered for immunoprecipitation was of around 300–400 bp. For each sample 100 μg of chromatin and 5 μg of the indicated antibodies (see Supplementary Table 1) or 20ul of anti-c-Myc magnetic beads (ThermoScientific, #88842) were used. All qPCRs analysis were performed using Power SYBR Green Master Mix (Applied Biosystems by ThermoFisher Scientific) and run in the QuantStudio 6-Flex (ThermoFisher Scientific). Primers used for qPCR analysis are reported in Supplementary Table 2 [12, 58]. ChIP analysis was performed using the percent of Input as method.

### Immunofluorescence

Cells were seeded on gelatin coated slides and left in culture for 24 or 48 h. After this time, cells were fixed in 3.7% formaldehyde for 15 min, followed by permeabilization with 0.25% Triton X-100 for 5 min at room temperature. Cells were blocked for 45 min in 3% BSA, 0.1% Tween-20 in 1× PBS and incubated with primary antibodies (as indicated in Supplementary Table 1) diluted in blocking solution at room temperature for 2 h. Cells were washed in 0.3% BSA, 0.1% Tween-20 in 1x PBS and incubated with secondary antibodies (Supplementary Table 3) for 1 h at room temperature. Slides were washed in 0.1% Tween-20 1XPBS, stained with DAPI (Sigma-Aldrich) and mounted with Mowiol (Millipore-Calbiochem). For the analysis of nuclear circularity images were acquired with a Zeiss LSM510 inverted confocal microscope (Carl Zeiss, Gottingen, Germany) and circularity index was calculated using an ImageJ plugin, Active contour (ABSnake).

### Polarity assay

Centrosome orientation assay was performed as described in Chang et al. [37] with same minor adaptations. Briefly, cells were plated on gelatin coated slides. After 48 h, when the cells were at 90% of confluence, were starved with serum-free medium. 48 h later, confluent monolayers were wounded with a 10-μl micropipette tip, and incubated for 30 min at 37 °C to allow them to recover. Next, cells were incubated with serum-free medium containing LPA (10 μM), or without LPA as negative control, and maintained at 37 °C for indicated times. Slides were then fixed and processed for immunofluorescence as described in immunofluorescence section (antibodies listed in Supplementary Table 1). Images were acquired with Leica DMIRE deconvolution microscope equipped with a Leica DFC 350FX camera and elaborated by a Leica LAS X software (Leica, Solms, Germany). Images analysis has been performed using ImageJ 1.46r. For each cell, a first line was drawn from the center of the nucleus to the wound edge and a second line was drawn from the center of the nucleus to the center of the centrosome. The angle formed between the two lines was measured.

### Single cell migration analyses

For random migration analysis, cells were seeded on 10 μg/ml fibronectin-coated 6 well plate and left to adhere in the incubator overnight. Cell nuclei were labeled for 20 min at 37 °C with Hoechst 34580 (Sigma-Aldrich) at 5 μg/ml or with NucBlue live cell stain (Thermofisher) according to the manufacturer’s instructions.

For 1D migration analysis, coverslips (25 mm) were micropatterned with 10 μm-wide lines as previously described [59] and coated with fibronectin (10 μg/ml). Cells expressing nuclear H2B-GFP were seeded on the coverslips and left to adhere in the incubator overnight.

Afterwards, the coverslips were mounted in 35 mm magnetic chambers (Chamlide) for imaging.

For both random and 1D analyses, cell migration was monitored with a 10× objective every 10 min for 24 h by fluorescence and transmission light, using an inverted wide-field motorized microscope (Olympus Scan^R) in a humidity- and temperature controlled environmental chamber (37 °C & 5% CO_2_ perfusion). Cell velocity, persistence and effective length were analyzed as previously described [59].

### 3D spheroids invasion assay

For 3D invasion assay, Cultrex 3D Spheroid Cell Invasion Assay kit (Trevigen) was used according to the manufacturer’s instructions. Briefly, cells spheroids were generated by plating 2500 cells with a specialized ECM^R^ in 96 well round bottom plates to drive spheroid formation. After 3 days spheroids were embedded in Invasion Matrix^R^ and photographed every 24 h at 4× magnification using a Nikon Eclipse TS100 microscope equipped with Infinity 1 digital camera. Invasive activity of each spheroid was quantified as the surface area of the invasive structure by using ImageJ.

### Spontaneous model of metastasis: orthotopic tumor implantation in mouse mammary fat pad

Female NSG mice (NOD.Cg-Prkdc^SCID^IL-2R null) (Charles River Laboratories, Calco, Italy) at 6 weeks of age were anesthetized with a combination of tiletamine–zolazepam (Telazol, Virbac, Carros, France) and xylazine (xylazine/Rompun BAYER) given intramuscularly at 2 mg/kg, and a small incision of less than 3 mm is made externally and caudally to the nipple. With the aid of micro-dissecting forceps, the ventralmost part of the fat pad is gently pulled out and exposed through the small incision.

LUC MDA-MB-231 or LUC MDA-MB-436 cells (1 × 10^6^) shSCR or shTRF2 were randomly injected using an insulin syringe with a 27 gauge niddle. Successful injection is confirmed by the swelling of the tissue. The small incision is sealed using absorbable suture (PolySorb^TM^ 5-0) [60]. Finally, mice were medicated with an orally administration of 0.5 mg/kg of Metacam (meloxicam) to control post-operative pain and inflammation. Real time tumor growth was monitored weekly using the IVIS Lumina II CCD camera system (PerkinElmer). Mice were injected intraperitoneally with 150 mg/Kg D-Luciferin (PerkinElmer) and imaged 10 min after luciferin injection. Imaging was performed at baseline and periodically (every 7–10 days) after tumor cell injection. Bioluminescence signals were determined by the number of photons and were acquired and analyzed using the Living image software version 4.3 (PerkinElmer). Primary tumors were surgically resected and fixed in formalin for IHC analysis. Mice were monitored periodically by Ivis imaging for metastases appearance until sacrifice when lungs were fixed in formalin and processed for IHC.

### Experimental model of metastasis: intravenously injection of tumor cells

Female NSG mice (NOD.Cg-Prkdc^SCID^IL-2R null) at 6 weeks of age were randomly injected in the tail vein with 2 × 10^5^ LUC MDA-MB-231 shSCR or shTRF2 cells in a volume of 200 μl PBS/mouse. Real time lung metastases were monitored weekly using the IVIS Lumina II CCD camera system (PerkinElmer). Mice were injected intraperitoneally with 150 mg/Kg D-Luciferin (PerkinElmer) and imaged 10 min after luciferin injection. Bioluminescence signals were determined by the number of photons and were acquired and analyzed using the Living image software version 4.3 (PerkinElmer). Mice were sacrificed and lungs were fixed in formalin for IHC analysis.

### Intravital imaging

All animal experiments were approved by the Animal Welfare Committee of the NKI, in accordance with national guidelines. All animals were maintained in the animal department of the NKI, housed in individually ventilated cage (IVC) systems under specific pathogen-free conditions and received food and water ad libitum. Tumor cells (1 × 10^6^) were injected in the right inguinal mammary gland of female NOD-scid Il2ry^null^B2m^null^ mice knockout mice at 10–20 weeks of age. Mice bearing tumors of ~250 mm3 were used for intravital microscopy. Mice were sedated using isoflurane inhalation anesthesia (1.5% to 2% isoflurance/air mixture). The imaging site was surgically exposed, and the mouse was placed with its head in a facemask within a custom designed imaging box. The isoflurane was introduced through the facemask, and ventilate by an outlet on the other side of the box. The temperature of the imaging box and microscope were constantly adjusted to keep the mice between 36 and 37 °C by a climate chamber that surrounds the whole stage of the microscope including the objectives. All images were acquired on an inverted Leica TCS SP8 multiphoton microscope. All images were collected in 12 bits with a 25X water immersion objective (HC FLUOTAR L N.A. 0.95 W VISIR 0.17 FWD 2.4. mm). All images were processes using ImageJ software and were smoothed (if necessary) and contracted linearly (if necessary). Images were corrected for XY and Z drift using custom-written software. Cell tracking analysis was performed using MTrackJ plugin for ImageJ [61].

### Tissue analyses (mouse)

#### Immunohistochemistry (IHC)

Formalin-fixed and paraffin embedded tissue blocks were sectioned (2 μm) and subjected to deparaffinization, rehydratation and antigen retrieval by PT Link (Dako Omnis), that allows the entire pretreatment process of tissue sections, low or high pH according to primary antibodies datasheet. Endogenous peroxidase was blocked for 10 min in Peroxidase-Blocking Solution (3% hydrogen peroxide in methanol, Dako Omnis) and then non-specific antibody binding was blocked for 20 min with Protein blocking buffer (Dako Omnis).

Sections were immunostained for 1 h at room temperature with primary antibodies (Supplementary Table 1) and then were covered with secondary antibody for 30 min at room temperature. The signal was developed by using DAB detection kit (Dako Omnis), then sections were counterstained with Harry’s modified Hematoxylin. Finally, slides were washed, dehydrated with increasing alcohol and xylene, and mounted with Eukitt (Sigma Aldrich). Immunostaining results were recorded as percentage of positive cells.

#### Tunel assay

Formalin-fixed and paraffin-embedded tissue blocks were sectioned, deparaffinized and rehydrated through a graded ethanol series. Detection of apoptotic cells by terminal deoxytransferase-mediated deoxy uridine nick end-labeling (TUNEL) assay was carried out utilizing In Situ Cell Death Detection POD Kit (Roche Molecular Biochemicals). Sections were stained according to the manufacturer’s protocol for paraffin-embedded tissues. Immunostaining results were recorded as percentage of positive cells.

#### H&E staining

Formalin-fixed and paraffin embedded tissue blocks were sectioned (3 μm), deparaffinized and rehydrated through decreasing alcohol, finally washed in distilled water. Sections were stained by immersing slides in hematoxylin for 3 min, washed with running water for a few minutes to allow stain to develop and then with distilled water. Slides were counterstained with Eosin for 3 min, dehydrated with alcohol and xylene and mounted with Eukitt (Sigma Aldrich). Results were recorded as metastatic area (μm²) or metastatic foci/section.

### TRF2 immunohistochemistry analysis in human breast cancer patients

TRF2 immunostaining was performed in a series of 30 primary triple-negative breast carcinomas (BC) and their corresponding metachronous metastases, 55 invasive breast carcinomas with different molecular subtypes (10 LuminalA BC, 10 LuminalB/HER2 negative BC, 10 LuminalB/HER2 positive BC, 9 HER2 subtype BC, 16 triple-negative subtype BC) and 50 benign breast lesions with their adjacent normal tissue (evaluable in 41 cases), surgically treated at the Regina Elena Cancer Institute (Rome, Italy) between 2001 and 2018. Formalin-fixed paraffin-embedded specimens were cut on SuperFrost Plus slides (Menzel-Gläser, Braunschweig, Germany). 3 μm thick formalin-fixed paraffin-embedded sections were stained with a Bond Polymer Refine Detection on an automated autostainer (BondTM Max, Leica Biosystems, Milan, Italy) with anti-TRF2 antibody listed in Supplementary Table 1. Diaminobenzidine (DAB) was used as chromogenic substrate. Immunostaining was evaluated by 2 biologists (SB, ADB) and TRF2 was assessed as positive when tumor cells showed immunoreaction in at least 10% neoplastic cells. Immunostaining results were recorded as IRS resulting from multiplication of TRF2 positive cell proportion score and TRF2 staining intensity score as described in Fedchenko et al. [62].

### TCGA dataset and bioinformatics analysis

Normalized TCGA-BRCA gene expression of tumor samples were obtained from Broad Institute TCGA Genome Data Analysis Center (http://gdac.broadinstitute.org/): Firehose stddata__2016_01_28. Broad Institute of MIT and Harvard. 10.7908/C11G0KM9.

Statistical significance of gene modulation between different subgroup of samples was assessed by Wilcoxon test. ANOVA test was performed for comparison of more than two groups. Significance was defined at the *p* < 0.05 level.

Overall survival (OS) were performed by using Kaplan-Meier analysis and the log-rank test was used to assess differences between curves. Patients with high and low signal intensity were defined by considering positive and negative *z*-score values, respectively.

The analyses were completely conducted with Matlab R2020b.

Lamin-B1 DamID-Seq data on HCT116 cell lines were downloaded from the 4DN Data portal (https://data.4dnucleome.org) and then intersected with TRF2 ChIP-seq data on HCT116 generated at our Institute. Common peaks were identified with the *mergePeaks* function available within the software for motif discovery and ChIP-Seq analysis Homer [63], using a distance of 500 bp.

### Statistical analysis

For all the presented data, statistical tests used, and *n* values are reported in the relative Figure legend. *P* values were calculated with GraphPad Prism 6. *P* values were indicated as followed *P ≤ 0.05; **P ≤ 0.01; ***P ≤ 0.001. Where not reported *P* value is not statistically significant.

## Supplementary information

Supplementary Information

Original Data File

## Data Availability

All data generated or analyzed during this study are available within the article and Supplementary Files or available from the authors upon request.
