# Supplementary material for: TRF2 interaction with nuclear envelope is required for cell polarization and metastasis in triple negative breast cancer
Source: Cell Death Dis. 2025 Mar 30;16(1):224. doi: 10.1038/s41419-025-07415-4 (PMC11955551; doi:10.1038/s41419-025-07415-4)
Supplement: Supplementary file 1 — Supplementary Information [file 41419_2025_7415_MOESM1_ESM.pdf]

TRF2 interaction with nuclear envelope is required for cell polarization and metastasis in triple negative breast cancer

Petti et al.

SUPPLEMENTARY INFORMATION:

- Supplementary Figure 1
- Supplementary Figure 2
- Supplementary Figure 3
- Supplementary Figure 4
- Supplementary Figure 5
- Supplementary Figure 6
- Supplementary Figure 7
- Supplementary Figure 8
- Supplementary Figure 9
- Supplementary Materials and Methods
- Supplementary Table 1
- Supplementary Table 2
- Supplementary Table 3
- Supplementary References

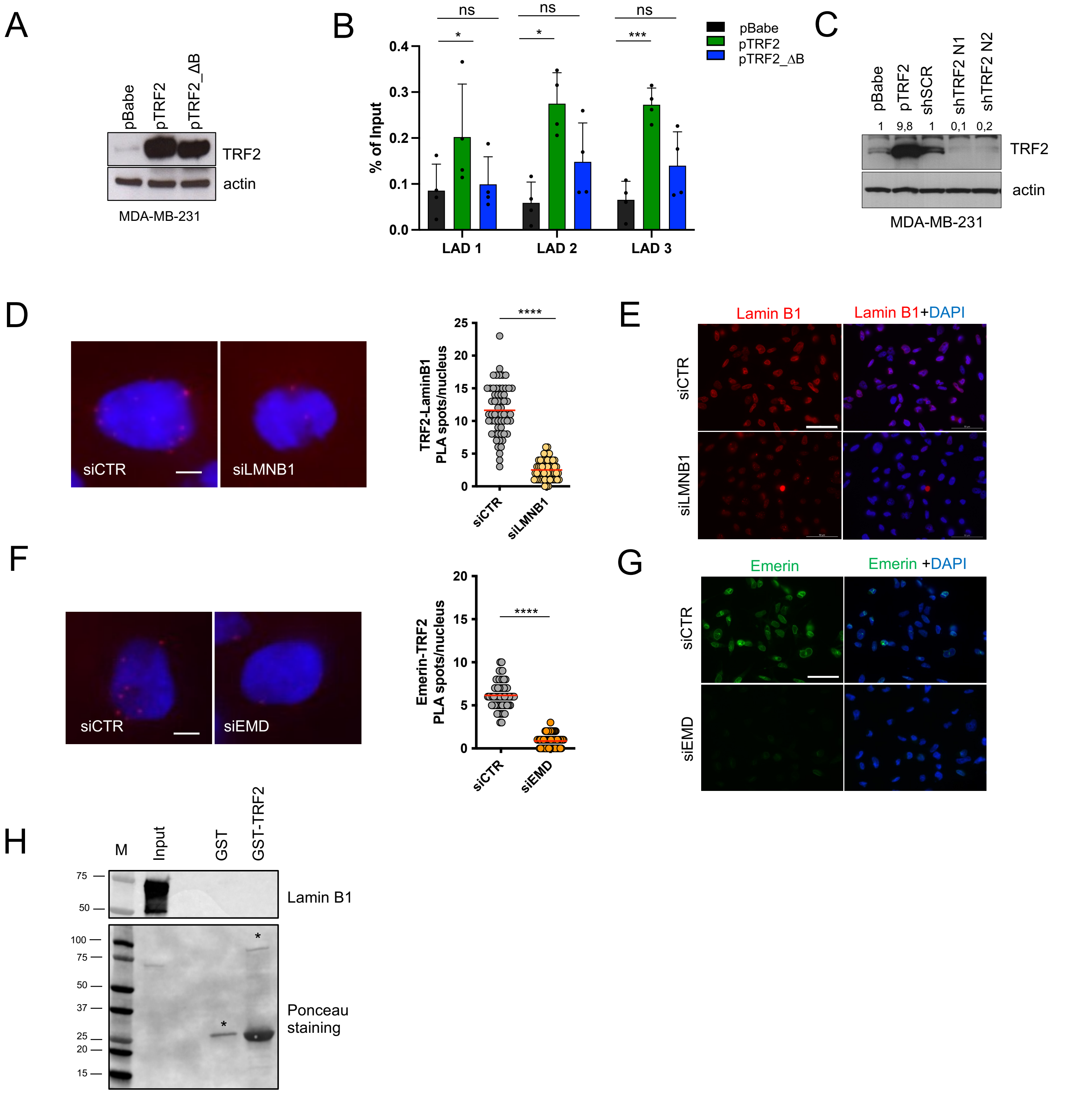

Supplementary Figure 1. TRF2 interacts with nuclear envelope components. A. MDA-MB-231 cells stable overexpressing TRF2 WT (pTRF2) or TRF2_ΔB (pTRF2_ΔB) deletion mutant and their control (pBabe) were subjected to Western blotting for TRF2 expression analysis. **B**. ChIP qPCR analysis shows the binding of myc-TRF2 WT (pTRF2) or myc-TRF2 ΔB (pTRF2_ ΔB) and relative control (pBabe) on the indicated LADs. **C.** Stable TRF2 overexpressing (pTRF2) or interfered (shTRF2 N1, shTRF2 N2) MDA-MB-231 cells and their control counterparts (pBabe/shSCR) were subjected to Western blotting for TRF2 expression analysis. Discrete values of quantitative TRF2 densitometric analysis are reported on the top of each blot; controls were set to 1. **D**. Proximity ligation assay with antibodies for TRF2 and Lamin B1 in MDA-MB-231 cells transiently silenced for Lamin B1 (as control of Lamin B1 antibody specificity). *Left panel*: representative images, scale bars, 50μm. *Right panel*: quantification of TRF2-LaminB1 PLA spots per nucleus (n=60 cells). **E.** Immunofluorescence with anti-Lamin B1 antibody to verify silencing efficiency in MDA-MB-231. **F.** Proximity ligation assay with antibodies for TRF2 and Emerin in MDA-MB-231 cells transiently silenced for Emerin (as control of Emerin antibody specificity). *Left panel*: representative images scale bars, 50μm. *Right panel*: quantification of TRF2-Emerin PLA spots per nucleus (n=60 cells). **G.** Immunofluorescence with anti-Emerin antibody to verify silencing efficiency in MDA-MB-231. **H.** *In vitro* GST pull-down assay with GST alone or GST-TRF2 in the presence of myc-Lamin B1 recombinant protein. Following the pull-down, the samples were analyzed by western blotting using specific anti-Lamin B1 antibody. Purified GST fusion proteins (indicated by the asterisks) were visualized by ponceau staining. For B, two-tailed *t student* test was used to calculate statistical significance. For D, F, Mann Whitney test was used to calculate statistical significance. *P<0.05, **P<0.01, ***P<0.001, ****P<0.0001.

Supplementary Figure 2

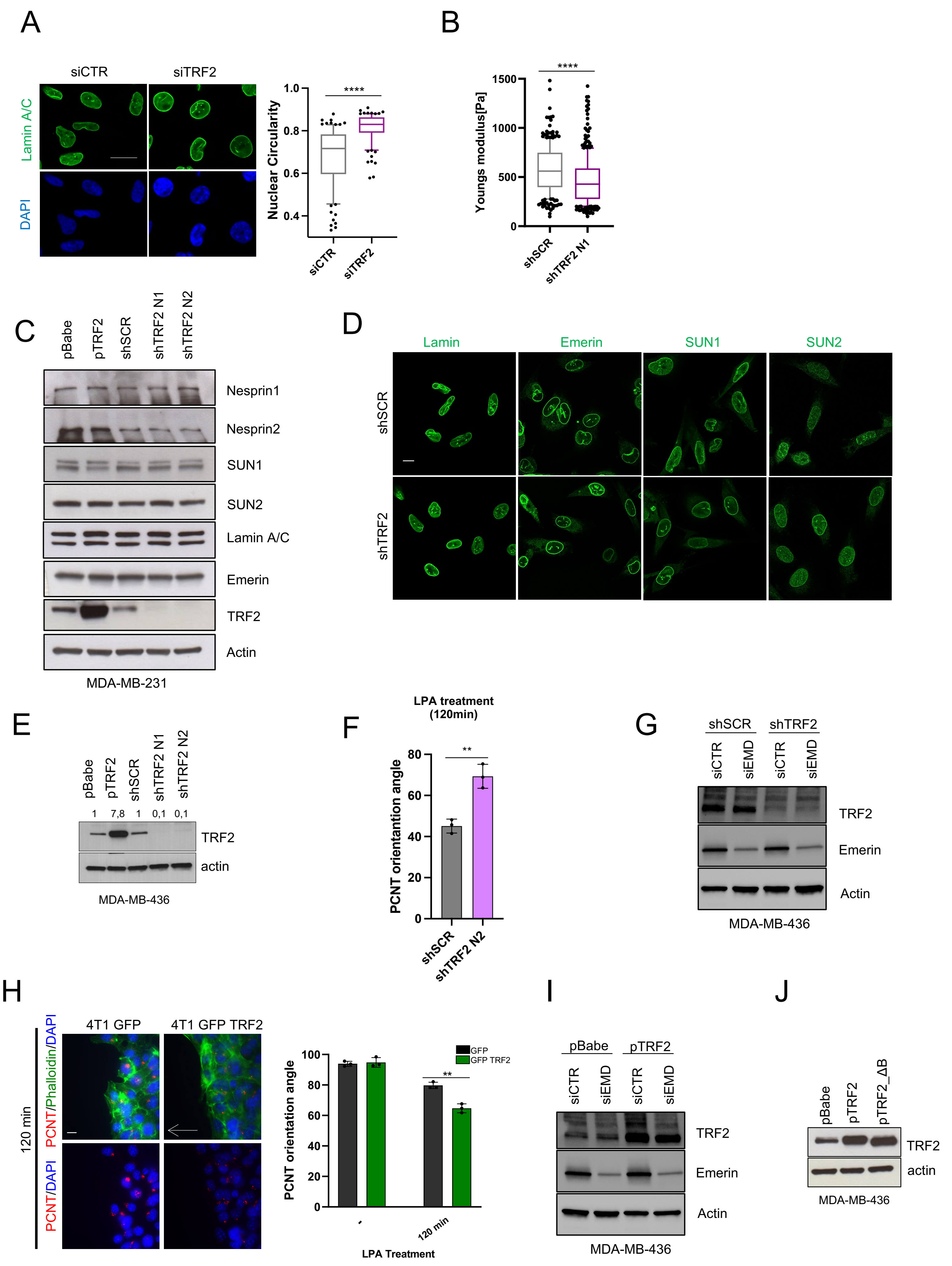

Supplementary Figure 2. Modulation of TRF2 expression impacts on nuclear circularity without affecting expression levels and localization of nuclear envelope components. A. Nuclear morphology analysis of MDA-MB-231 cells transiently transfected with TRF2 specific siRNA or with a non-targeting control siRNA. *Left panel*: representative images of anti-Lamin A/C staining and DAPI (scale bar, 20 μm). *Righ panel*: Box-and-whisker plot (10-90 percentile) of nuclear circularity index (n=90 cells). **B.** Box-and-whisker plots (10-90 percentile) of cellular stiffness measurement of TRF2-depleted MDA-MB-231 cells compared to control cells using AFM (shSCR n= 268 mesurements, shTRF2 N1 n=326 measurements; N=2 independent experiments). **C**. Western blotting analysis of MDA-MB-231 cells stable overexpressing or depleted for TRF2 with indicated markers. **D**. Immunofluorescence analysis of MDA-MB-231 stable depleted for TRF2 and relative control with indicated antibodies, scale bar, 20μm. **E**. Stable TRF2 overexpressing (pTRF2) or interfered (shTRF2 N1, shTRF2 N2) MDA-MB-436 cells and their control counterparts (pBabe/shSCR) were subjected to Western blotting for TRF2 expression analysis. Discrete values of quantitative TRF2 densitometric analysis are reported on the top of each blot; controls were set to 1. **F.** Stable TRF2 interfered (shTRF2 N2) MDA-MB-436 cells and their respective control (shSCR) were processed as described in Figure 2C. Quantification of centrosome orientation angles upon LPA treatment (120min) are shown. **G.** Stable TRF2 interfered (shTRF2 N1) MDA-MB-436 cells and their respective control (shSCR) were transfected with indicated siRNA and subjected to western blotting for TRF2 and Emerin expression analysis. **H.** Stable TRF2 overexpressing 4T1 cells (GFP-TRF2) and respective control cells (GFP) were processed as described in Figure 2C. *Left panel*, Representative images at 120 min are shown. Scale bar, 10 μm. *Right panel*, quantification of centrosome orientation angle in untreated or LPA treated cells in indicated experimental conditions. **I.** Stable TRF2 overexpressing MDA-MB-436 cells (pTRF2) and respective control cells (pBabe) were transfected with indicated siRNA and subjected to western blotting for TRF2 and Emerin expression analysis. **J**. MDA-MB-436 cells stable overexpressing TRF2 WT (pTRF2) or TRF2_ΔB (pTRF2_ΔB) deletion mutant and their control (pBabe) were subjected to Western blotting for TRF2 expression analysis. For A-B, Mann Whitney test was used to calculate statistical significance. For F, H, data are the mean ± SD (n = 3 independent experiments), two-tailed *t student* test was used for statistical significance. *P<0.05, **P<0.01, ***P<0.001, ****P<0.0001.

Supplementary Figure 3

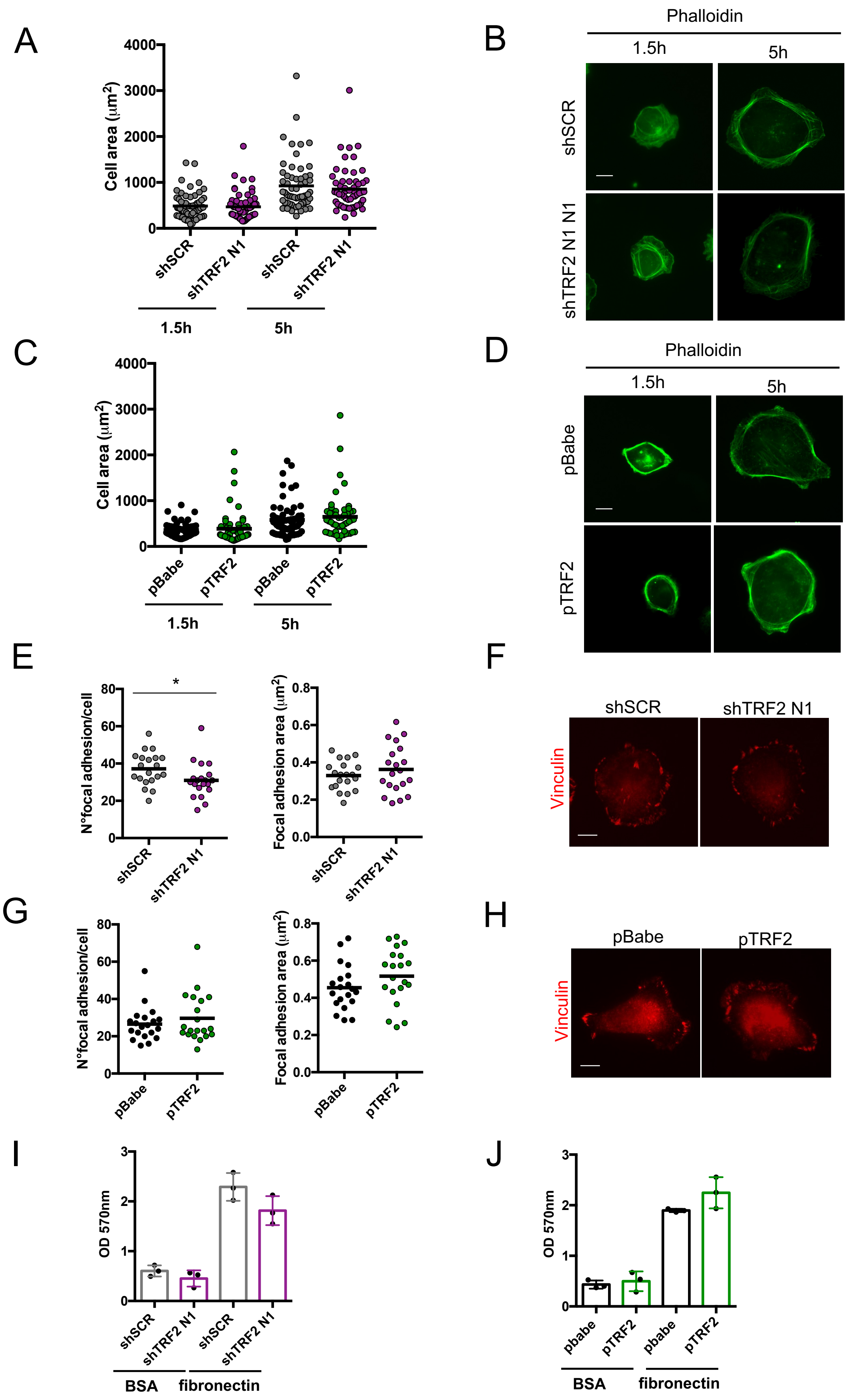

Supplementary Figure 3. Modulation of TRF2 expression does not affect cell spreading and adhesion. A. Stable TRF2 depleted MDA-MB-231 cells and their respective control (shSCR) were seeded on fibronectin-coated slides, fixed at indicated times and stained with Phalloidin. Data are cell area measurements (μm^2). Black lines indicate mean. At least 50 cells have been analysed for experimental condition. **B**. Representative images of A. are shown, scale bar, 20μm. **C**. Stable TRF2 overexpressing MDA-MB-231 cells (pTRF2) and respective control cells (pBabe) were processed as described in A. Data are cell area measurements (μm^2). Black lines indicate mean. At least 50 cells have been analyzed for experimental condition. **D**. Representative images of C. are shown, scale bar, 20μm. **E**. Stable TRF2 depleted MDA-MB-231 cells and their respective control (shSCR) were seeded on fibronectin-coated slides, fixed after 5 hours and subjected to immunofluorescence with anti-Vinculin antibody. Data are focal adhesion number/cell (left panel) and focal adhesion area measurements (μm^2)/cell (right panel). Black lines indicate mean. At least 20 cells and 600 focal adhesions have been analyzed for experimental condition. **F**. Representative images of E. are shown, scale bar, 10μm. **G**. Stable TRF2 overexpressing MDA-MB-231 cells (pTRF2) and respective control cells (pBabe) were processed as described in E. Data are focal adhesion number/cell (left panel) and focal adhesion area measurements (μm^2)/cell (right panel). Black lines indicate mean. At least 20 cells and 600 focal adhesions have been analyzed for experimental condition. **H**. Representative images of G. are shown, scale bar, 10μm. **I-J**. Stable TRF2 interfered (shTRF2 N1) (**I**) or overexpressing (pTRF2) MDA-MB-231 cells (**J**) and their control counterparts (shSCR/pBabe) were subjected to adhesion assay. Cells were plated on fibronectin or BSA as negative control. Data are means ± SD (*n* = 3 independent experiments). For all the data, two-tailed *t student* test was used for statistical significance, $^*P<0.05$, $^{**}P<0.01$, $^{***}P<0.001$, $^{****}P<0.0001$.

Supplementary Figure 4

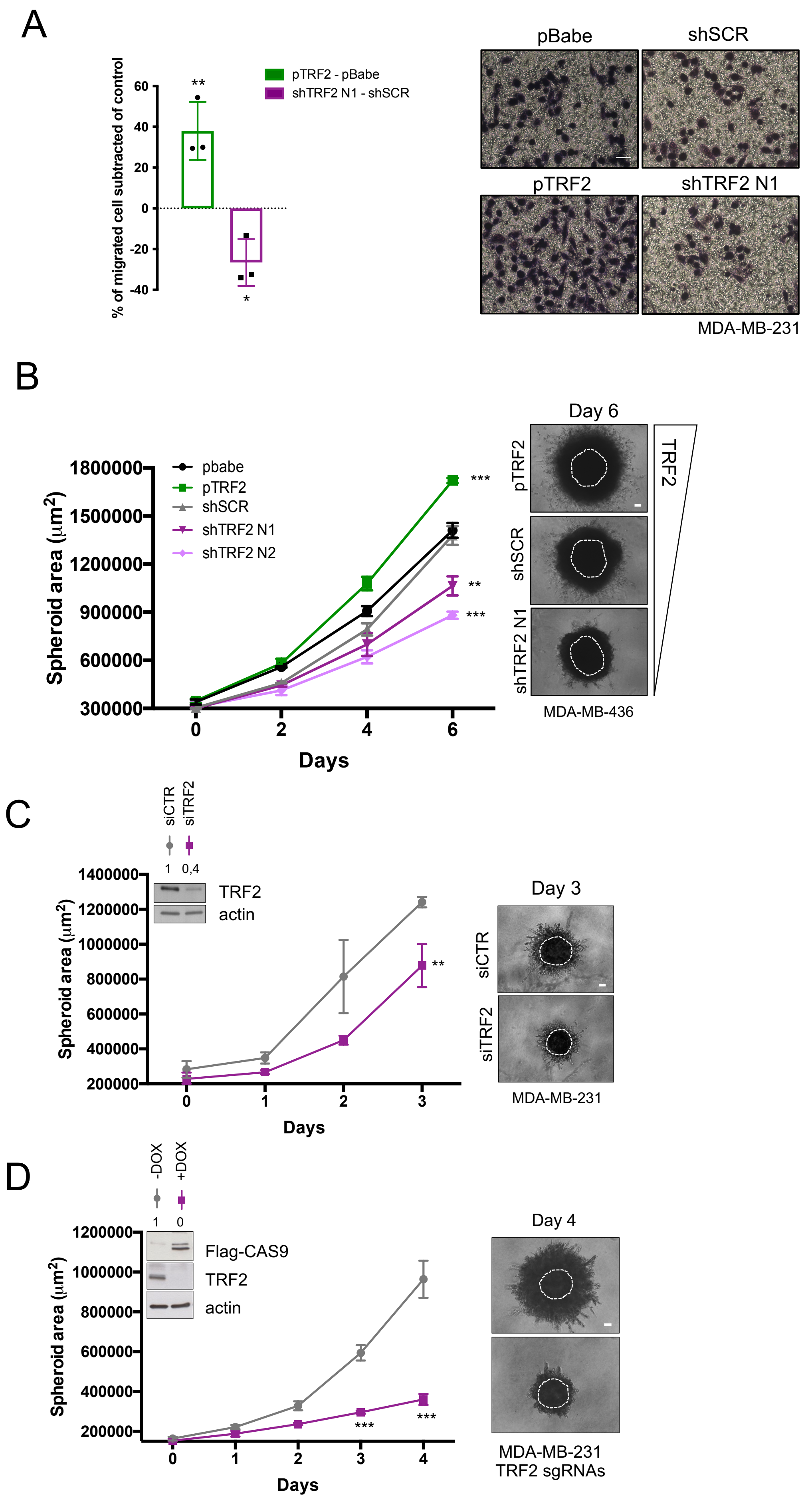

Supplementary Figure 4. The pro-migratory role of TRF2 is conserved in different cell models. A. *Left panel*: stable MDA-MB-231 cell lines overexpressing (pTRF2) or interfered for TRF2 (shTRF2 N1) and their control counterparts (pBabe/shSCR) were subjected to transwell migration assay. Data are the mean ± SD of migrated cells (%) subtracted of control (pTRF2-pBabe; shTRF2 N1-shSCR) (*n* = 3 independent experiments, fifteen fields analyzed for each experiment); controls set to 100%. *Right panel*: representative images of crystal violet dye staining of the lower chambers.Scale bar, 200µm. **B.** Stable TRF2 overexpressing (pTRF2) or interfered (shTRF2 N1, shTRF2 N2) MDA-MB-436 cells and their control counterparts (pBabe/shSCR) were subjected to 3D spheroid invasion assay. For 3D spheroid invasion assay, data are the mean of spheroids area ± SD (*n* = 3 independent experiments). *Right panel*: phase contrast images of representative spheroids for the indicated experimental conditions at day 6 are shown. Dashed circle indicates the size of spheroids at day 0. Scale bars, 100µm. **C**. *Left panel*: MDA-MB-231 cells were transiently transfected with TRF2 specific siRNA or with a non-targeting control siRNA and subjected to Western blotting for TRF2 expression analysis and to 3D spheroid invasion assay as described in Figure 1B. For Western blotting, discrete values of quantitative TRF2 densitometric analysis are reported on the top of each blot; si-control was set to 1. For 3D spheroid invasion assay, data are the mean of spheroids area ± SD (*n* = 3 independent experiments). *Right panel*: phase contrast images of representative spheroids at day 3 are shown. Dashed circle indicates the size of spheroids at day 0. Scale bars, 100µm. **D**. *Left panel*: Doxycycline-inducible CRISPR/CAS9 TRF2 knockout MDA-MB-231 cells were cultured in the absence (-) or in the presence (+) of doxycycline for 3 days and subjected to Western Blotting analysis with the indicated antibodies and to 3D spheroid invasion assay as described in Figure 1B. For Western blotting, discrete values of TRF2 quantitative densitometric analysis are reported on the top of each blot; - doxy was set to 1. For 3D spheroid invasion assay data are the mean of spheroids area ± SD (*n* = 3 independent experiments). *Right panel*: phase contrast images of representative spheroids at day 6 are shown. Dashed circle indicates the size of spheroids at day 0. Scale bars, 100µm. For A-D, two-tailed *t student* test was used to calculate statistical significance. *P<0.05, **P<0.01, ***P<0.001.

Supplementary Figure 5

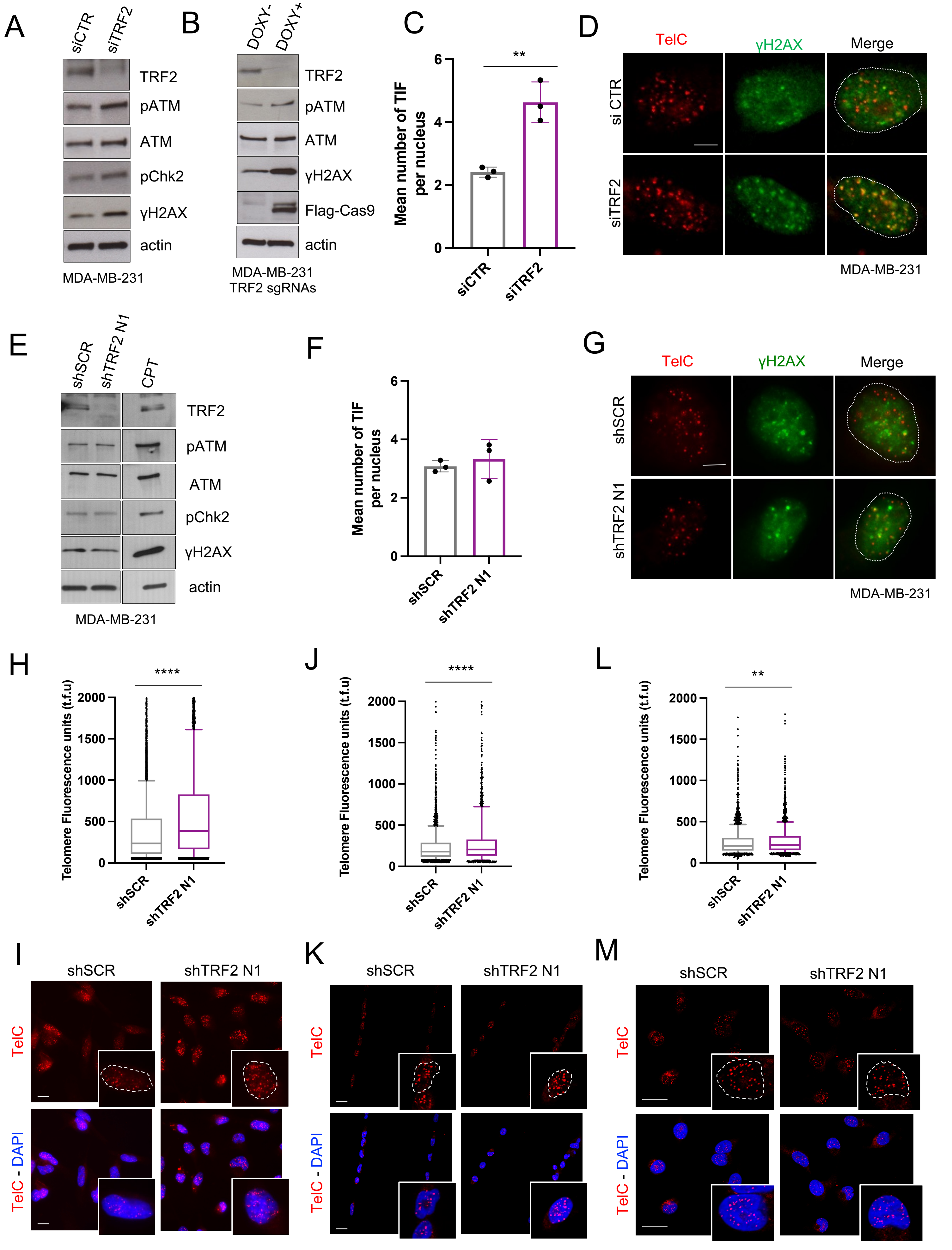

Supplementary Figure 5. DNA damage response analysis upon TRF2 modulation. A. Global DNA damage analysis upon RNAi-mediated silencing of TRF2. Indicated DNA damage markers were analysed by Western blotting. **B.** Global DNA damage activation analysis upon inducible CRISPR/Cas9 TRF2 KO. Indicated DNA damage markers were analysed by Western blotting. **C**. Telomeric DNA damage analysis by FISH with telomeric probe (TelC) combined with γH2AX immunofluorescence in indicated samples. Data are the mean number of colocalizations (TIF) per nucleus ± SD (*N* = 3 independent experiments, at least 60 nuclei analyzed per experimental condition). **D**. Representative images of C. Scale bar, 5μm. **E**. Global DNA damage activation analysis of stable TRF2 interfered (shTRF2 N1) MDA-MB-231 cells and their control counterpart (shSCR). Indicated DNA damage markers were analysed by Western blotting. Camptothecin treatment (0.5 μM, 24hrs) was used as positive control. **F**. Telomeric DNA damage analysis by telomeric FISH combined with γH2AX immunofluorescence in indicated samples. Data are the mean number of colocalizations (TIF) per nucleus ± SD (N = 3 independent experiments, at least 60 nuclei analyzed per experimental condition). **G**. Representative images of F. Scale bar, 5μm. **H.** Fluorescence intensity quantification of telomeric signals in stable TRF2 interfered (shTRF2 N1) MDA-MB-231 cells and their control counterpart (shSCR)($n \geq 200$ nuclei) plated in 2D migration conditions and processed for Telo-FISH. Box-and-whisker plots (10-90 percentile) where middle line represents the median value of arbitrary telomere fluorescence units (a.f.u.) **I.** Representative images of H. Scale bar, 20 μm. **J.** Fluorescence intensity quantification of telomeric signals in stable TRF2 interfered (shTRF2 N1) MDA-MB-231 cells and their control counterpart (shSCR)($n \geq 200$ nuclei) plated on fibronectin micropattern lines (1D migration conditions) and processed for Telo-FISH. Box-and-whisker plots (10-90 percentile) where middle line represents the median value of arbitrary telomere fluorescence units (a.f.u.). **K.** Representative images of J. Scale bar, 20 μm. **L.** Fluorescence intensity quantification of telomeric signals in stable TRF2 interfered (shTRF2 N1) MDA-MB-231 cells and their control counterpart (shSCR)($n \geq 100$ nuclei), upon dissociation at the end of 3D spheroid invasion assay. Box-and-whisker plots (10-90 percentile) where middle line represents the median value of arbitrary telomere fluorescence units (a.f.u.) **M.** Representative images of L. Scale bar, 20 μm. For C, F, statistical significance was calculated by two-tailed *t student* test. For H, J, L, statistical significance was calculated by Mann–Whitney test.

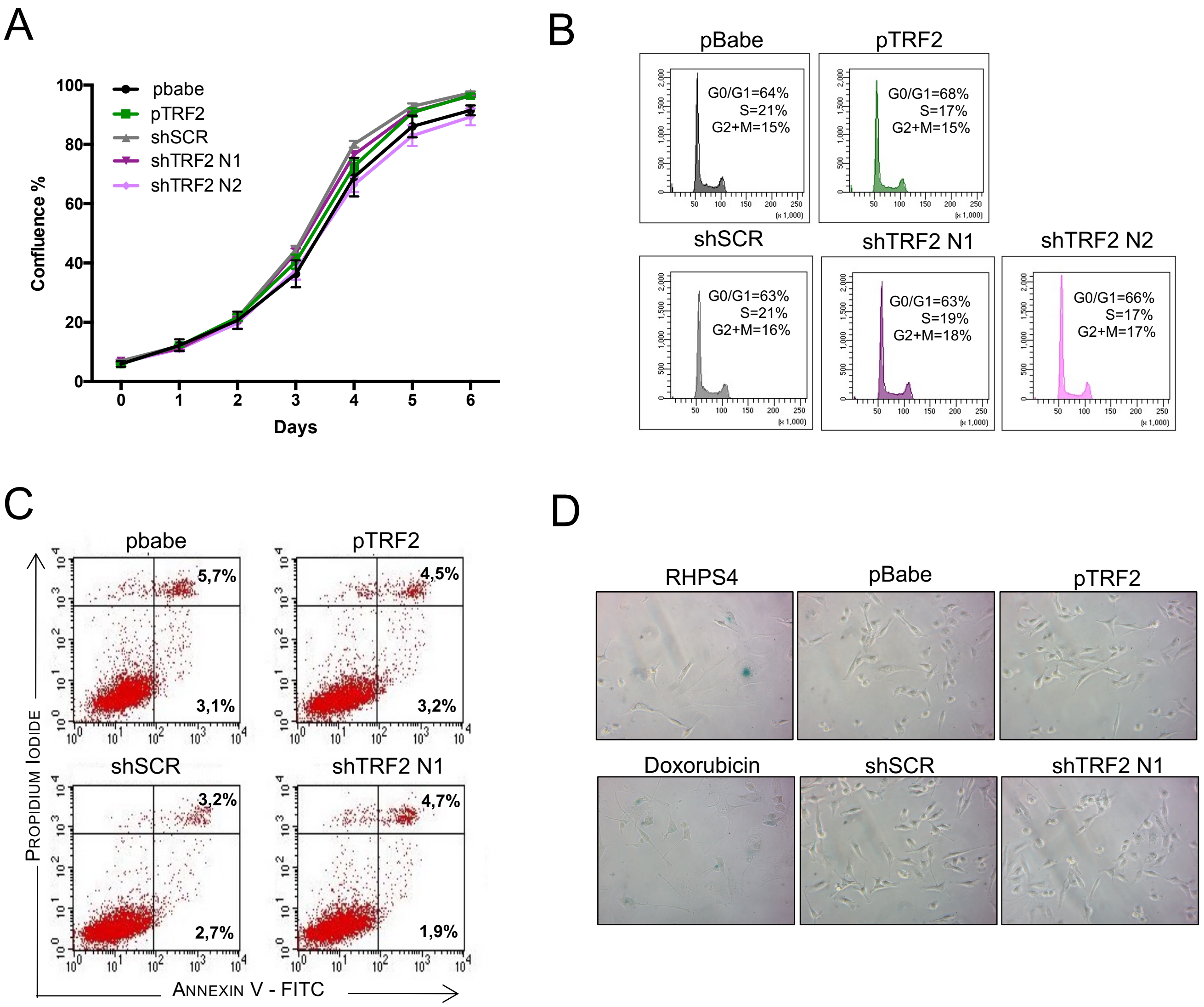

Supplementary Figure 6. Modulation of TRF2 expression does not affect cell growth and viability. A. Stable TRF2 overexpressing (pTRF2) or interfered (shTRF2 N1, shTRF2 N2) MDA-MB-231 cells and their control counterparts (pBabe/shSCR) were seeded and cell proliferation was monitored through 6 days by IncuCyte[R] System. Data are the mean of confluence (%) ± SD (*n* = 3 independent experiments). **B**. Cells from A were processed for FACS analysis to evaluate cell cycle by Propidium Iodide (PI) staining. **C**. Stable TRF2 overexpressing (pTRF2) or interfered (shTRF2 N1) MDA-MB-231 cells and their control counterparts (pBabe/shSCR) were assayed for apoptosis activation by annexin V expression analysis. **D**. Cytochemical staining of senescence associated β-gal activity of cells in C. As positive controls, MDA-MB-231 cells were treated with G4 ligand RHPS4 (10μM 72hrs) and Doxorubicine (100nM 72hrs). Representative light microscope images are shown.

Supplementary Figure 7

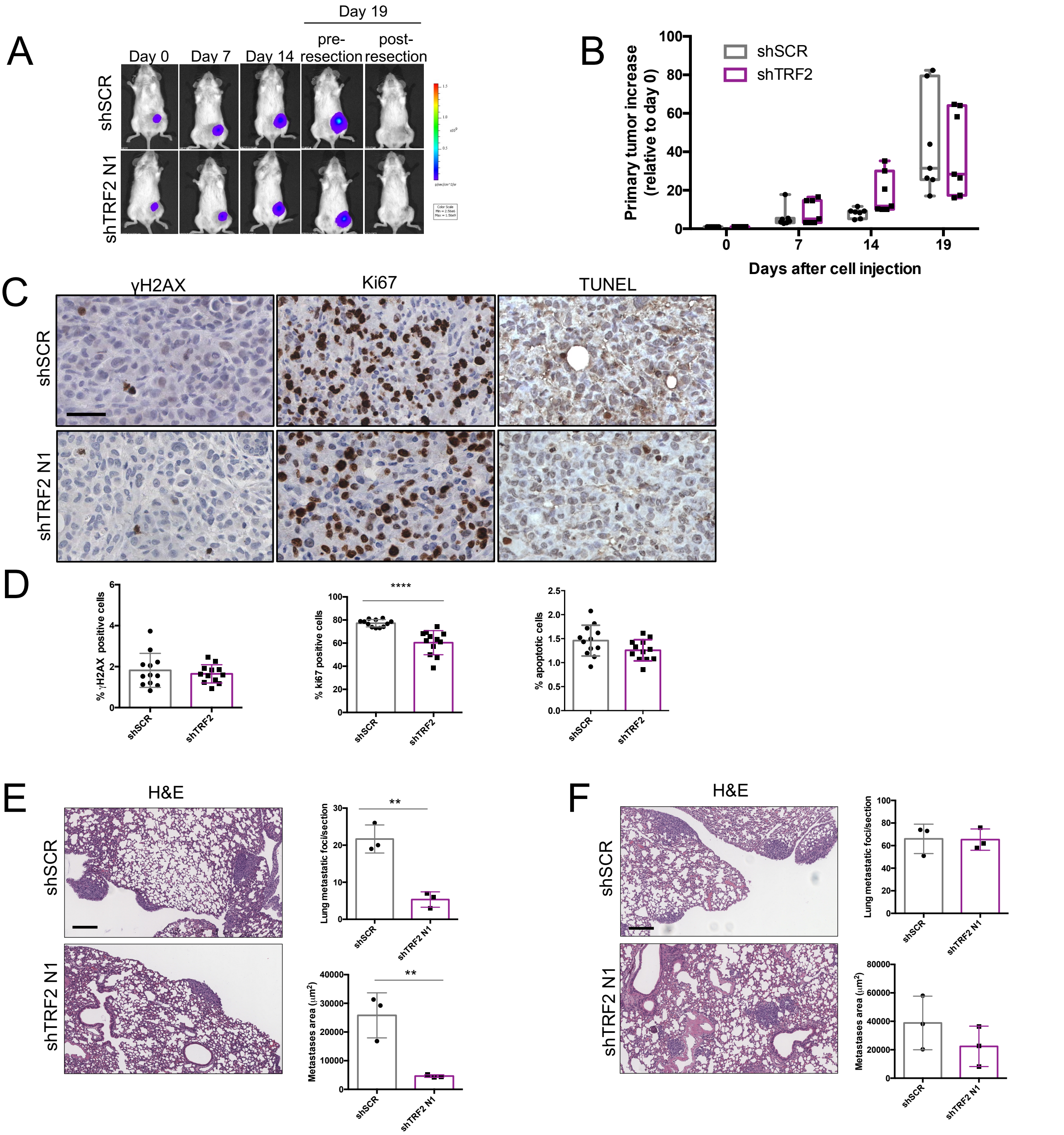

Supplementary Figure 7. TRF2 knockdown does not affect primary tumor growth and reduces formation of spontaneous metastasis. A. Luminescent MDA-MB-231 shSCR and shTRF2 N1 cells were orthotopically injected in the mammary gland of female NSG mice. Representative images of primary tumor growth at indicated times. **B**. Box plot representing primary tumors bioluminescence intensity. shSCR (N=7), shTRF2 (N=7). **C.** Immunoistochemical analysis of primary tumors derived from orthotopic injection of LUC shSCR or shTRF2 N1 MDA-MB-231 cell lines. Analyzed markers are indicated (γH2AX, Ki67, TUNEL ASSAY). Representative images are shown. Scale bars, 30μm. **D**. Quantification of C. Data are the percentage of positive cells ± SD. Twelve fields derived from three animals for condition were analyzed. **E.** *Left panel*, representative images of hematoxylin&eosin staining of lung sections from spontaneous metastasis experiment. Scale bars, 200 μm. *Right panel*, quantification of the number or area of metastatic foci. **F**. *Left panel*, representative images of hematoxylin&eosin staining of lung sections from experimental metastasis experiment. Scale bars, 200 μm. *Right panel*, quantification of the number or area of metastatic foci per section. Two-tailed *t student* was used to calculate statistical significance. *P<0.05, **P<0.01, ***P<0.001, ****P<0.0001.

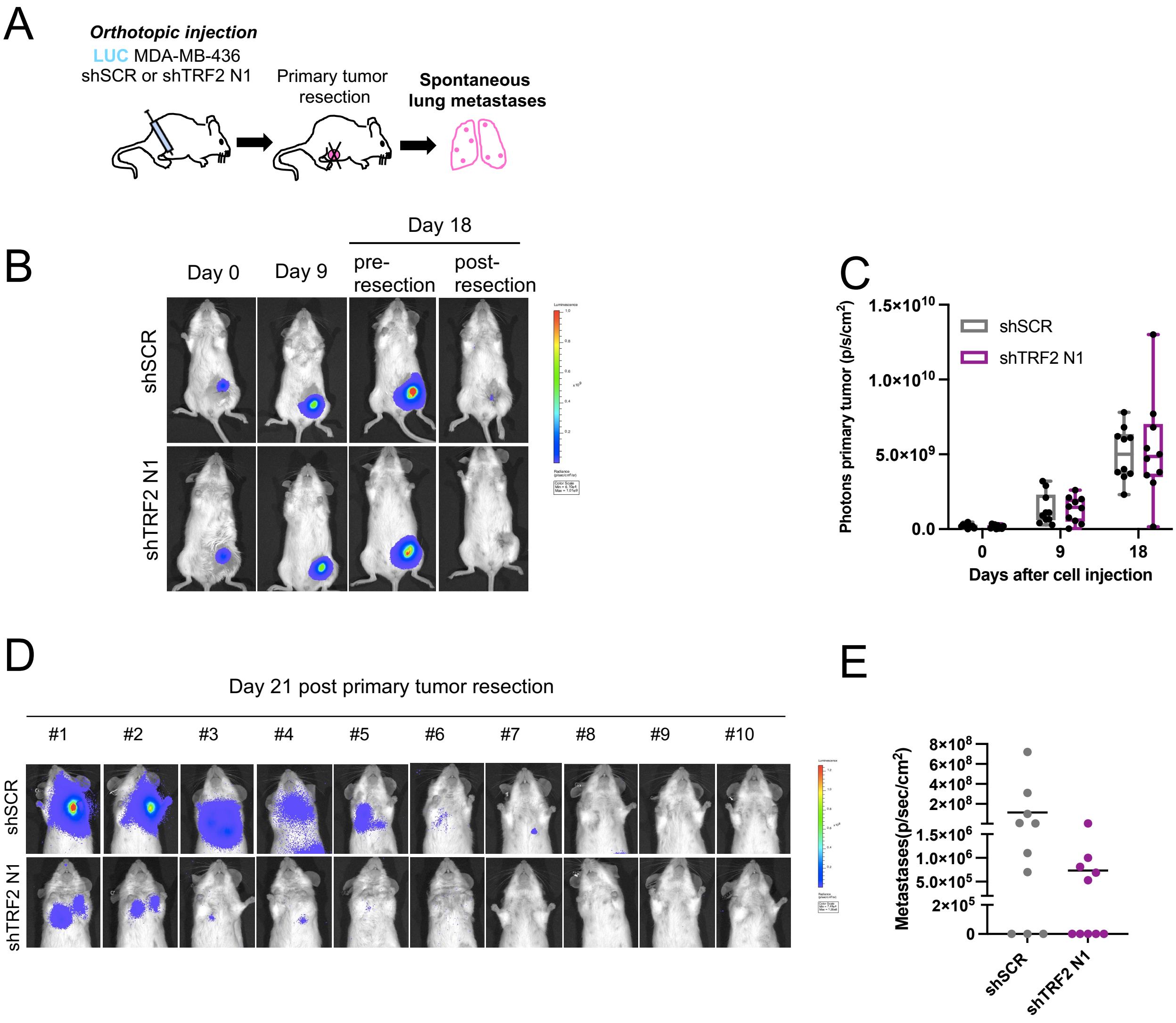

Supplementary Figure 8. TRF2 knockdown reduces formation of spontaneous metastases induced by orthotopic injection of MDA-MB-436 cells. A. Schematic representation of spontaneous metastasis experiment. Luminescent MDA-MB-436 shSCR and shTRF2 N1 were injected in the mammary gland of female NSG mice. Primary tumor was resected at day 18 and spontaneous lung metastases formation was monitored by bioluminescence imaging followed by *ex vivo* analyses. **B.** Representative images of primary tumor growth at indicated times. **C.** Box plot representing primary tumors bioluminescence intensity. shSCR (N=10), shTRF2 N1 (N=10). **D**. Representative images of all mice at day 21 post primary tumor resection. **E**. Box plot representing lung metastases bioluminescence intensity; shSCR (N=10), shTRF2 (N=10).

Supplementary Figure 9

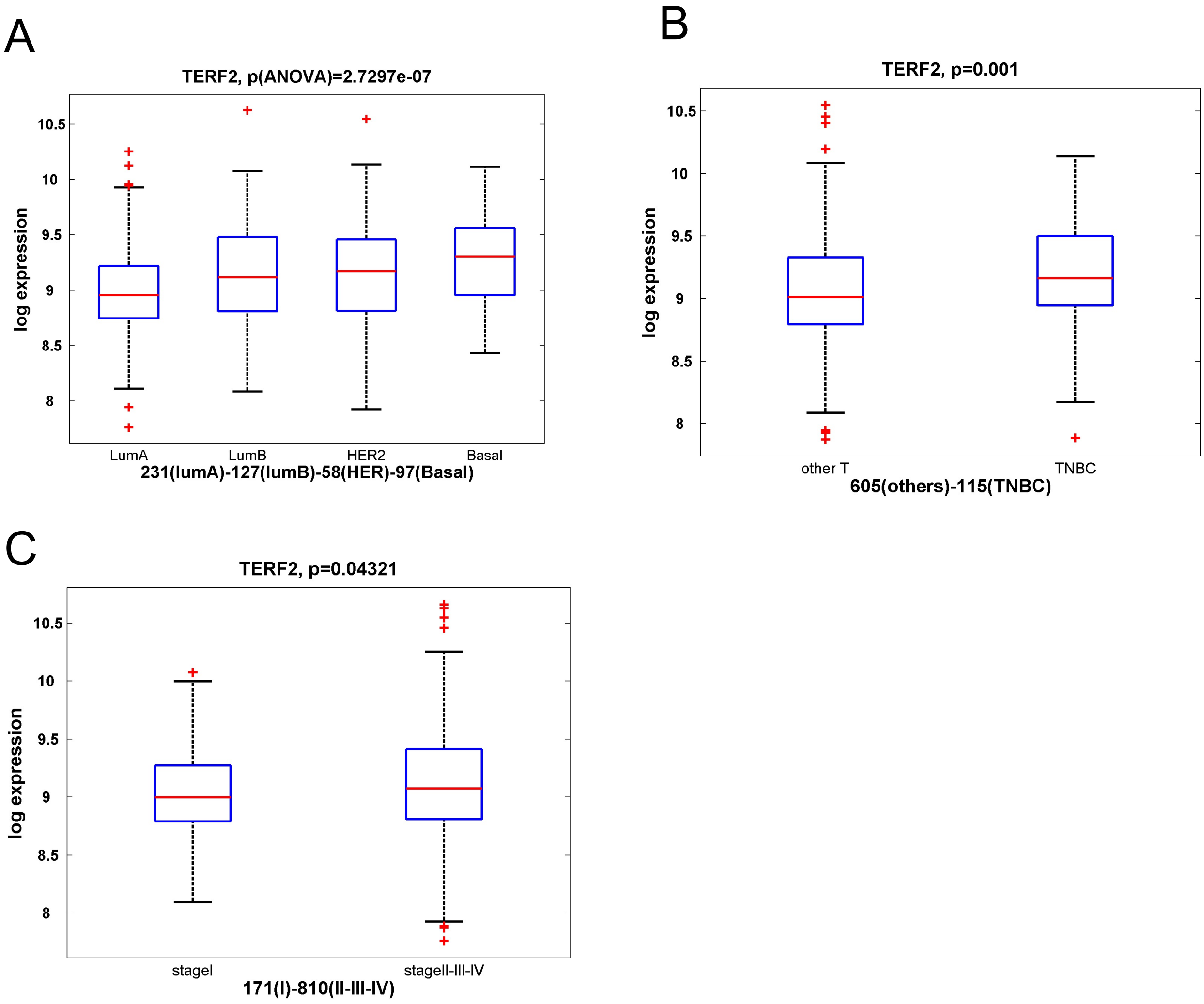

Supplementary Figure 9. TRF2 expression positively correlates with breast cancer aggressiveness. A. TRF2 mRNA expression evaluated on BC patients from TCGA dataset stratified on the basis of molecular BC subtypes. Luminal A, N=231; Luminal B, N=127; HER2+, N=58; Basal, N=97. Statistical significance was calculated by one-way ANOVA (****P<0.0001). **B**. TRF2 expression evaluated on BC patients from TCGA dataset stratified on the basis of the presence of progesterone, estrogen and HER2+ receptors. Patients with positivity at least to one receptor (others), N=605. Patients negative to all three receptors (TNBC), N=115; Statistical significance was calculated by one-way ANOVA (*P<0.05). **C**. TRF2 mRNA expression evaluated on stage **I** (N=171) compared to stages **II**-**III**-**IV** (N=810) BC patients from TCGA dataset. Statistical significance was calculated by one-way ANOVA (*P<0.05).

Supplementary Materials and Methods

AFM indentation method

A modified AFM tip (NovaScan, USA) attached with 10 μm diameter bead was used to indent the center of the cell, in correspondence of nucleus. The spring constant of the AFM tip cantilever is 0.03 N/m. AFM indentation loading rate is 0.5 Hz with a ramp size of 3 μm. AFM indention force was set at a threshold of 2 nN. The data points below 0.5 μm indentation depth were used to calculate Young's modulus, using the Hertz model shown below:

$$F = \frac{4}{3}\frac{E}{(1-\nu^2)}\sqrt{R\delta^3}$$

where F is the indentation force, E is the Young's modulus to be determined, v is the Poisson's ratio, R is the radius of the spherical bead, and δ is indentation depth. The cell was assumed incompressible and a Poisson's ratio of 0.5 was used.

Cell spreading and focal adhesion analysis

For spreading assay (3x104) cells were seeded on fibronectin coated slides and fixed after 1,5 and 5 hours with 3.7% formaldehyde for 15 minutes. Cell permeabilization and staining were performed following the immunofluorescence protocol previously described. The analysis was carried out by measuring the area of the cells with ImageJ Software.

For focal adhesion analysis (3x104) cells were seeded on fibronectin coated slides, fixed after 5 hours and subjected to immunofluorescence. For analysis, focal adhesion number/cell and focal adhesion area were measured using ImageJ Software.

Adhesion Assay

24-well plates were coated with BSA 1% or fibronectin 0,5% (Sigma Aldrich) at 37 °C overnight and blocked with BSA 1% for 1 hour. Cells were washed with PBS and $2x10^5$ cells, resuspended in PBS, were plated on each coated well and incubated at 37 °C. Nonadherent cells were removed by washing with PBS. Cells were fixed in 4% formaldehyde (PFA) for 15 minutes, washed 3 times with PBS and stained with crystal violet solution (0.5% in 20% methanol) for 30 minutes. Cells were measured in a spectrophotometer at 570 nm after solubilization in acetic acid.

Transwell migration assay

Cells were starved for 24 hours, then harvested in 5% BSA serum free medium, centrifuged and resuspended in serum free medium. Boyden Chamber consisting of transwell filter inserts with 8 µm size polycarbonate membrane (Corning-Costar,Lowell MA) placed in a 24-well plate were used. Cells ($9x10^4$) were placed in the upper chambers, while the lower chambers were filled with 10% FBS medium. After 6 hours, inserts were removed, and inner side was wiped with cotton swaps. Migrated cells were stained using Diff-Quick kit (Merz-Dade, Switzerland). After letting the inserts dry, pictures were acquired with 20X objective of Nikon Eclipse TS100 microscope equipped with Infinity 1 digital camera and cells per field were counted.

Immunofluorescence combined with telomere DNA FISH

Cells were seeded on gelatin coated slides and left in culture for 24 hours. After this time, cells were fixed in 4% formaldehyde for 15 minutes, followed by permeabilization with 0.1% Triton X-100 for 7 minutes at room temperature. Cells were blocked for 1 hour with 3% BSA

in 1X PBS and incubated with primary antibody (as indicated **in Supplementary Table 1**) diluted in blocking solution at 4ºC overnight. The next day, after two washes of 5 minutes with 0.05% Triton X-100 in 1x PBS, cells were incubated with secondary antibody (**Supplementary Table 3**) diluted in blocking solution for 1 hour room temperature. Slides were washed 2 times with 1X PBS for 7 minutes and Telomere DNA FISH was carried out as previously described [1]. Cells were subjected to consecutive fixations with 4% formaldehyde (PFA), then were dehydrated with increasing alcohols and allowed to dry at room temperature. Samples, to which the telomere probe solution (Cy3-labeled telomeric probe, Panagene) was added, were denatured for 3 minutes at 80ºC and incubated for 2 hours in humid chamber. After a series of washes with FISH solution (70% formamide, 10 mM Tris pH 7.2, 0.1% BSA), samples were incubated with DAPI, then dehydrated with 70%, 90%, and 100% ethanol and mounted with Mowiol. Nuclei showing at least three telomere-γH2AX colocalization events were considered for the quantification.

Quantitative telomeric FISH

Cells plated under 2D random migration conditions, cells plated on fibronectin micropatterned lines as in 1D migration and cells resulting from dispase dissociation (Sigma Aldrich,1mg/ml) [2] of spheroids following 3D invasion were subjected to standard FISH of telomeric DNA, as previously described [3]. Briefly, cells were dehydrated with alcohols, air-dried and hybridized with a Cy3-labeled telomeric probe (Panagene), denatured at 80 °C for 3 minutes, then incubated at room temperature in a humid chamber for 2 hours. Slides were then washed with FISH solution (70% formamide, 10 mM Tris pH 7.2, 0.1% BSA), finally stained with DAPI (Sigma-Aldrich) and mounted with Mowiol (Millipore-Calbiochem). Fluorescence signals were acquired at 63x magnification using a Zeiss LSM510 inverted confocal microscope (Carl Zeiss, Gottingen, Germany). The intensity of telomere signals was analyzed using the IOD spot analysis software (TFL-TELO).

Growth curve

Cells ($5x10^4$) were plated in each well of a 6-well plate and cell proliferation was monitored through 6 days using an Incucyte Live-Cell Imager (PHCBi Panasonic). Confluence of the cultures was measured using Incucyte software.

Cell senescence

Senescence was evaluated by β-galactosidase staining kit (Cell Signaling Technology) according to the manufacturer's instructions.

FACS analyses

Cell cycle analysis was performed by flow cytometry using a FACSCelesta (BD Biosciences, San Jose, CA, USA). Briefly, adherent cells (2×10^5) were fixed and resuspended in a solution containing propidium iodide at a concentration of 50 µg/ml and RNase at a final concentration of 75 kU/mL, incubated for 30 minutes in the dark.

Apoptosis was evaluated by annexin V versus PI assay, as described in [4]. Briefly, cells were harvested, suspended in annexin-binding buffer (1×10^6 cells/ml), incubated with fluorescein isothiocyanate-annexin V (annexin-FITC) and PI (Molecular Probes) for 15 minutes at room temperature in the dark, and then immediately analyzed by flow cytometry using a FACScan (BD Biosciences).

For each experiment, 20 000 events were collected using excitation/emission wavelengths of 488/525 and analyzed with CELLQuest and FACS Diva Software (BD Biosciences).

Supplementary Table 1: primary antibodies

Antibodies	Company	DILUTION WB	DILUTION IF	DILUTION IHC	DILUTION ChIP
**Mouse anti-TRF2 (4A794)**	Millipore, 05-521	1:1000	1:1500	1:500	
**Rabbit anti-TRF2**	Novus Biologicals, 110-57130				5ug
**Rabbit anti-γH2AX (BLR053F)**	Bethyl Laboratories, A700-053			1:500	
**Mouse anti-γH2AX (JBW301)**	Millipore, 05-636	1:1000	1:500		
**Rabbit anti-ATM, D2E2**	Cell Signaling Technology, 2873	1:1000			
**Rabbit anti-pATM, S1981**	Cell signaling Technology, 13050	1:1000			
**Mouse anti-Ki67 (MIB-1)**	Dako Omnis, GA626			Ready-to-use	
**Mouse anti-CK (AE1/AE3)**	Dako Omnis, M3515			1:50	
**Mouse anti-Lamin A/C (636)**	Santa Cruz Biotechnology, sc-7292	1:1000	1:500		
**Rabbit anti-Lamin B1**	Abcam, 16048	1:5000	1:500		5ug
**Mouse anti-Emerin (H-12)**	Santa Cruz Biotechnology, sc-25284	1:1000	1:200		
**Rabbit anti-SUN1**	Novus Biologicals, NBP1-87396	1:1000	1:200		
**Rabbit anti-SUN2 (EPR6557)**	Abcam, ab124916	1:2000	1:200		
**Mouse anti-β-Actin (AC-74)**	Sigma Aldrich, A2228	1:10000			
**Rabbit anti-PCNT**	Abcam, 4448		1:500		
**Vinculin (VIN-11-5)**	Sigma Aldrich, V4505		1:100		
**Purified Rabbit IgG**	Bethyl Laboratories, P120-101				5ug

Supplementary Table 2: ChIP qPCR-primers

	Forward primer	Reverse primer
SCN10A[5]	GGAAGAGGGTGAGGGAAAGC	TGTCACCAGTCCTTCCATGC
Subtel Chr1[6]	GCTGTATTGCAGGGTTCAACT	GGGTGTCATGTGTGCATTAGGA
LAD1	CATTTTGAGCTCCTTGGGGC	GTGCTCTGTCCAAACCAAGG
LAD2	GGGTCCTCTGCATGTGATCT	AGAAGGGTACAAGGTGCTGT
LAD3	GCAGGCCAGAATTCAAGTCC	CCAACATCAGGGAACAGTGC

Supplementary Table 3: Immunofluorescence secondary antibodies

Antibodies	Company
Goat anti-rabbit Alexafluor 488	Cell signaling Technology
Goat anti-mouse Alexafluor 488	Cell signaling Technology
Goat anti-rabbit Alexafluor 555	Cell signaling Technology
Goat anti-mouse Alexafluor 555	Cell signaling Technology
Alexa Fluor 488 phalloidin	Life Technologies

Supplementary References

1 Petti E, Buemi V, Zappone A, Schillaci O, Broccia PV, Dinami R *et al.* SFPQ and NONO suppress RNA:DNA-hybrid-related telomere instability. *Nat Commun* 2019; **10**.

2 Bahmad HF, Cheaito K, Chalhoub RM, Hadadeh O, Monzer A, Ballout F *et al.* Sphere-Formation Assay: Three-Dimensional in vitro Culturing of Prostate Cancer Stem/Progenitor Sphere-Forming Cells. *Front Oncol* 2018; **8**. doi:10.3389/fonc.2018.00347.

3 Rizzo A, Maresca C, D'Angelo C, Porru M, Di Vito S, Salvati E *et al.* Drug repositioning strategy for the identification of novel telomere-damaging agents: A role for <scp>NAMPT</scp> inhibitors. *Aging Cell* 2023; **22**. doi:10.1111/acel.13944.

4 Biroccio A, Benassi B, Filomeni G, Amodei S, Marchini S, Chiorino G *et al.* Glutathione influences c-Myc-induced apoptosis in M14 human melanoma cells. *Journal of Biological Chemistry* 2002; **277**: 43763–43770.

5 Lund EG, Duband-Goulet I, Oldenburg A, Buendia B, Collas P. Distinct features of lamin A-interacting chromatin domains mapped by ChIP-sequencing from sonicated or micrococcal nuclease-digested chromatin. *Nucleus* 2015; **6**: 30–39.

6 Dinami R, Petti E, Porru M, Rizzo A, Ganci F, Sacconi A *et al.* TRF2 cooperates with CTCF for controlling the oncomiR-193b-3p in colorectal cancer. *Cancer Lett* 2022; **533**: 215607.
