# Supplementary figures and images for: TRF2 interaction with nuclear envelope is required for cell polarization and metastasis in triple negative breast cancer
Source: Cell Death Dis. 2025 Mar 30;16(1):224. doi: 10.1038/s41419-025-07415-4 (PMC11955551; doi:10.1038/s41419-025-07415-4)

**B**

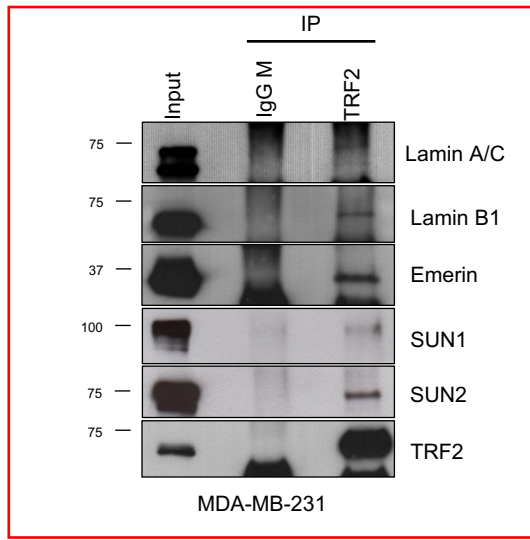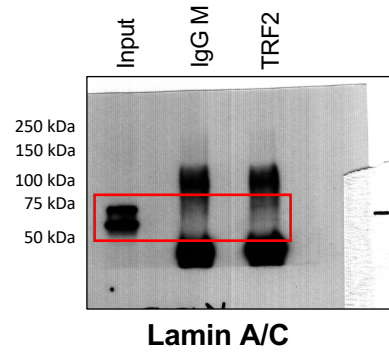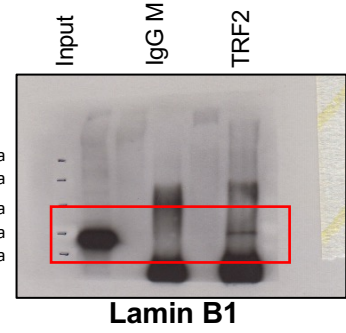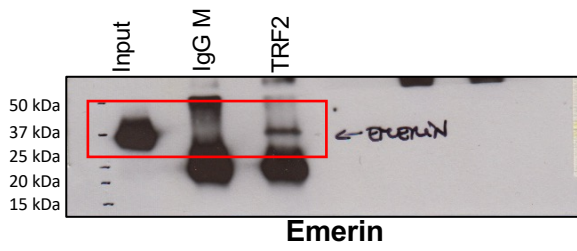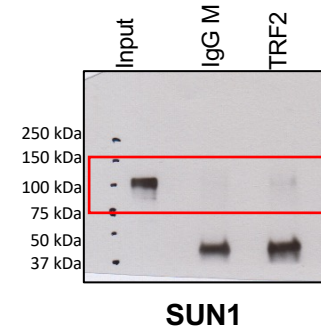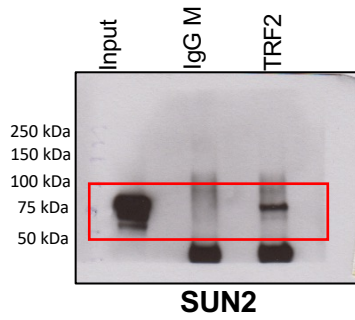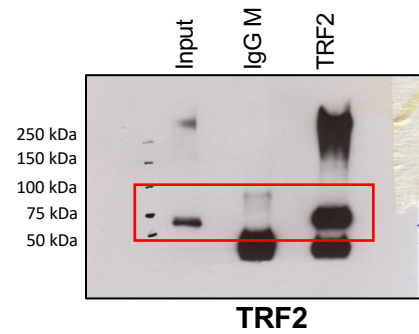

**D**

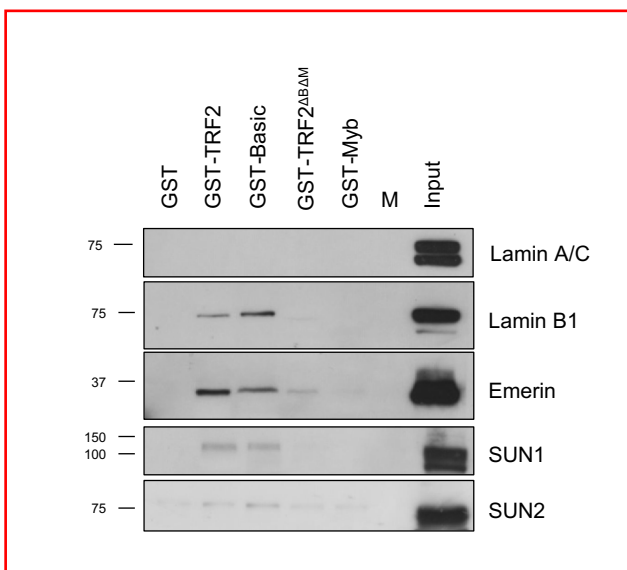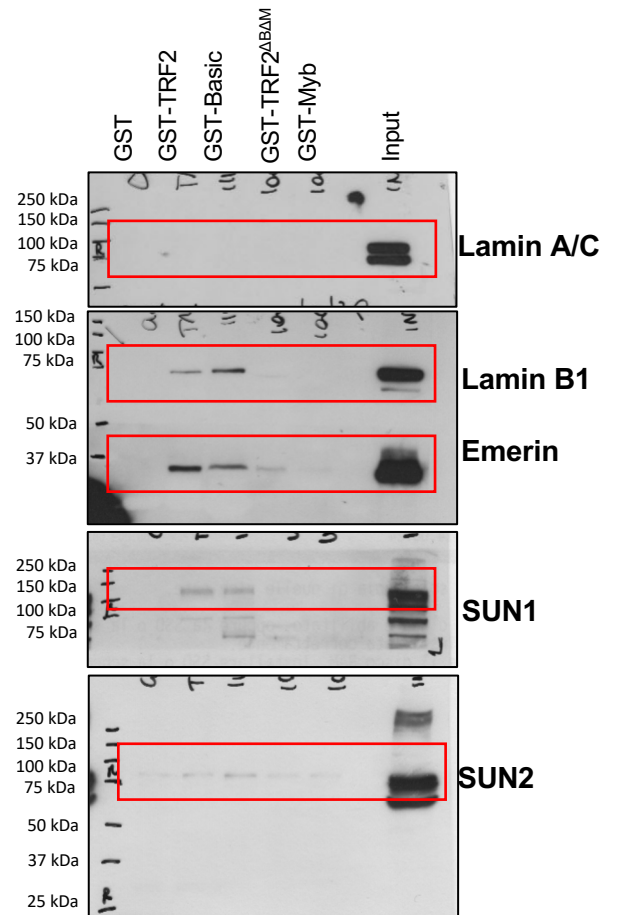

G

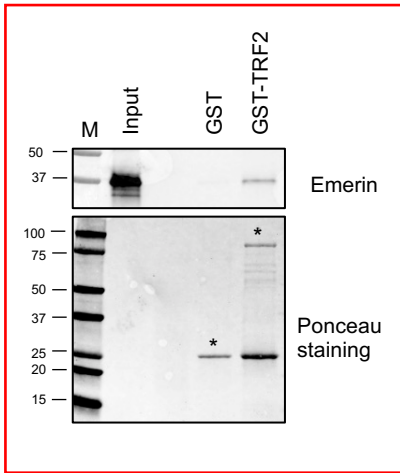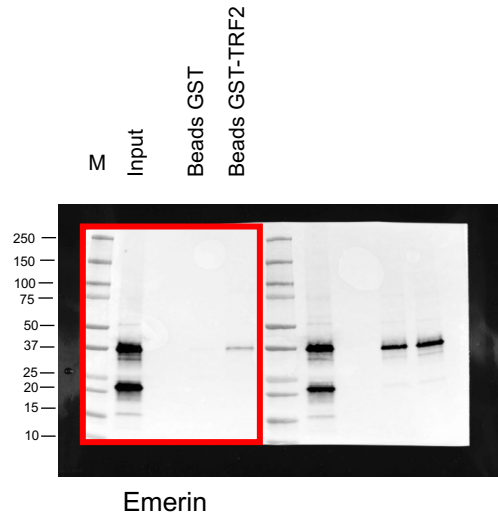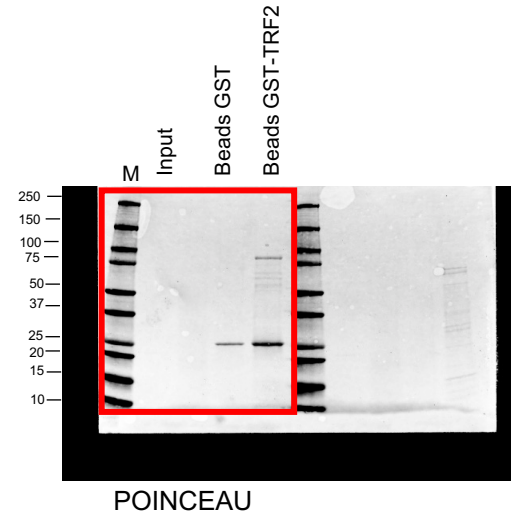

H

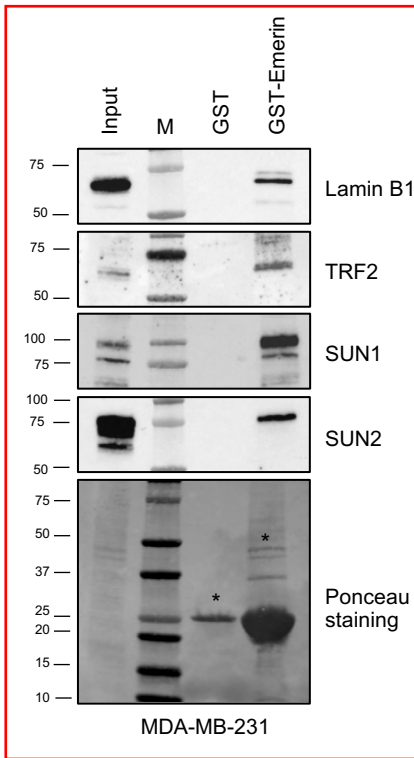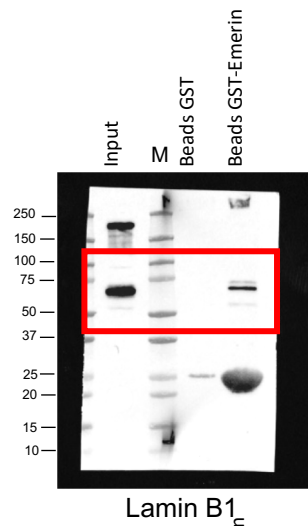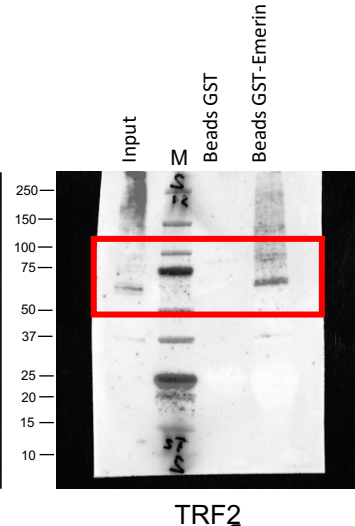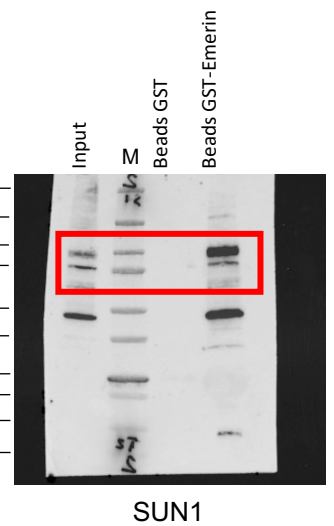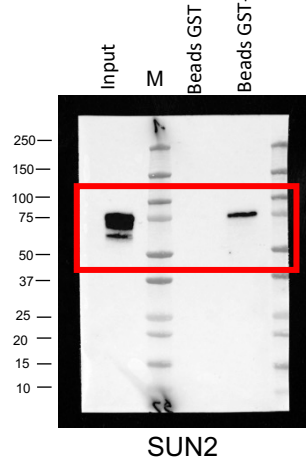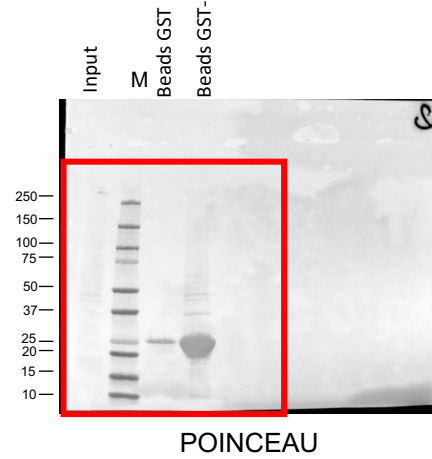

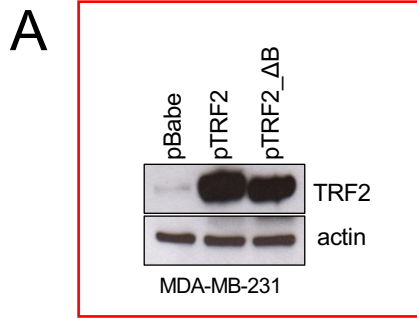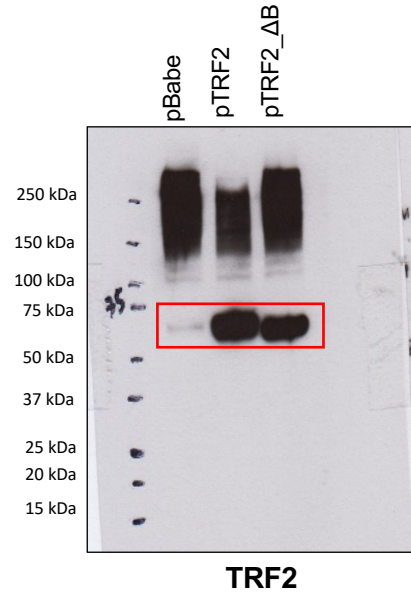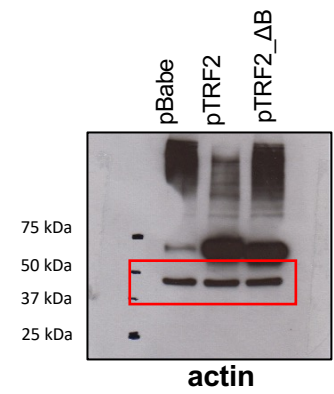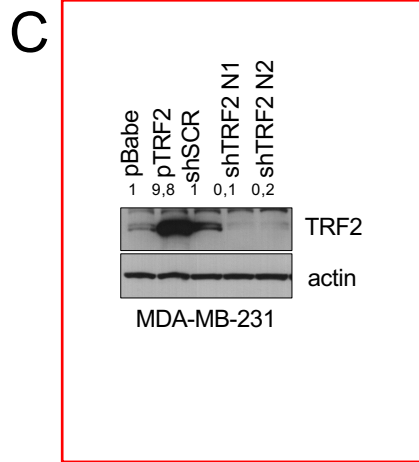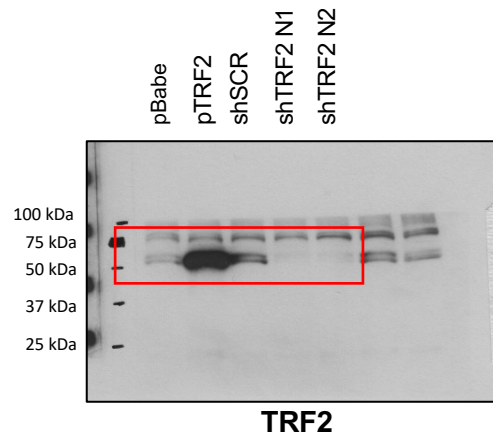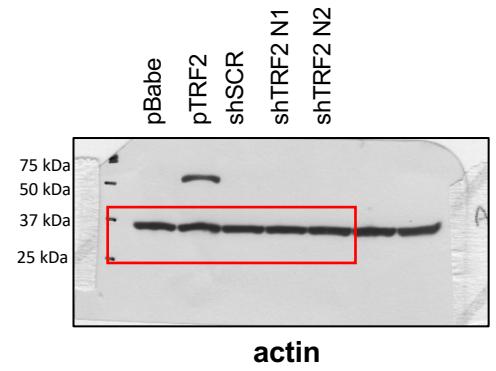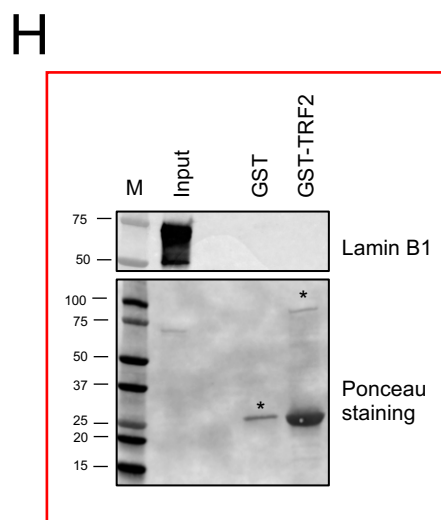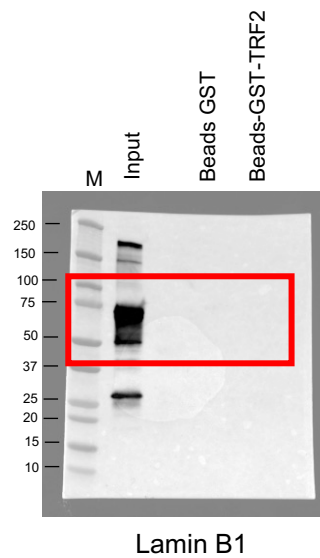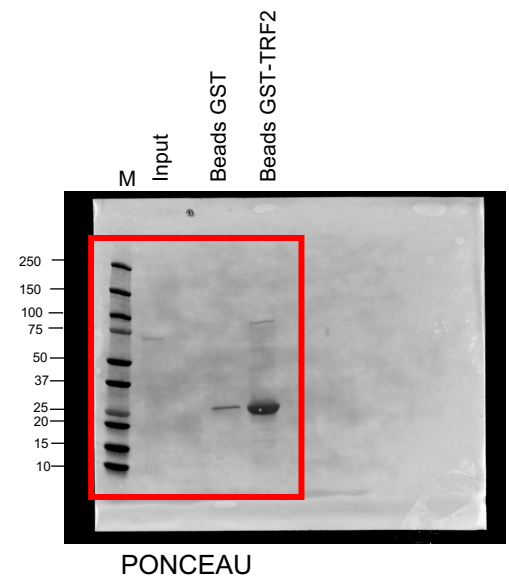

Supplementary Figure 2

C

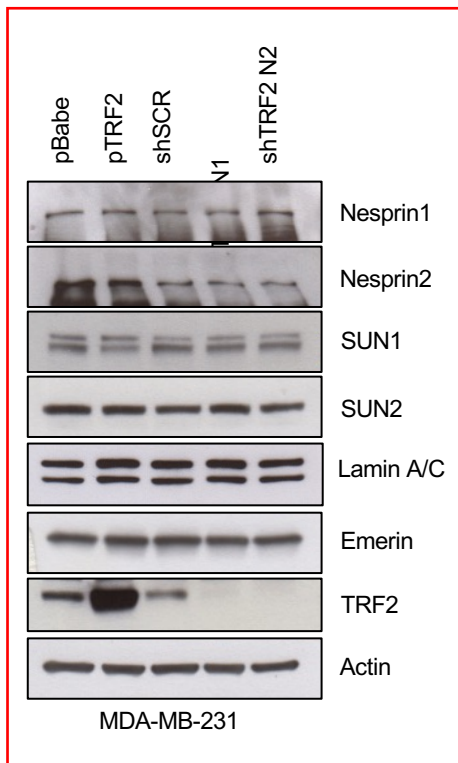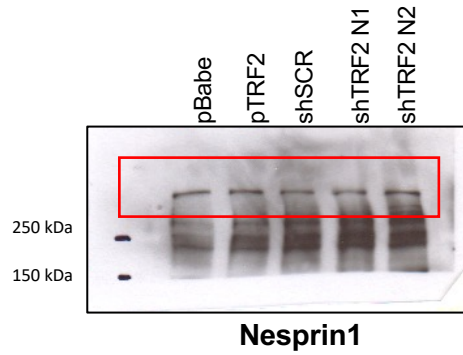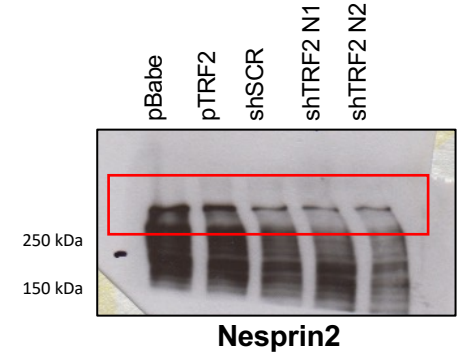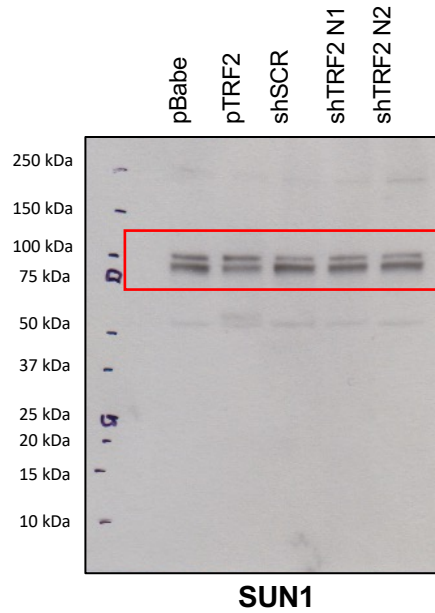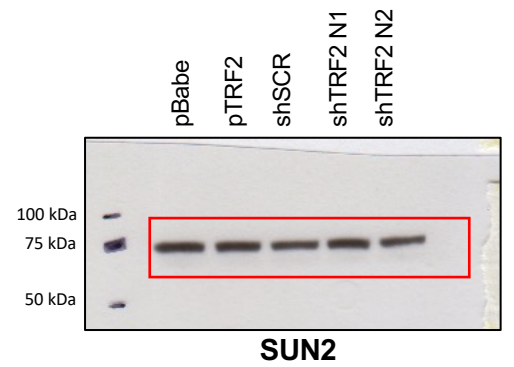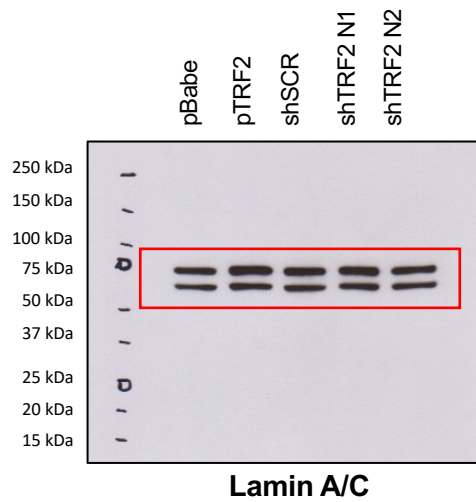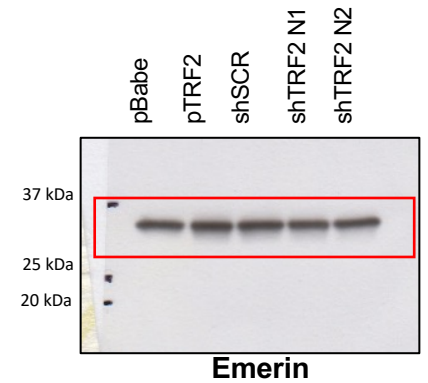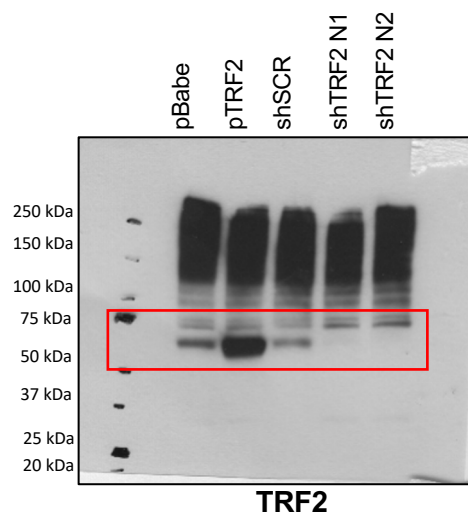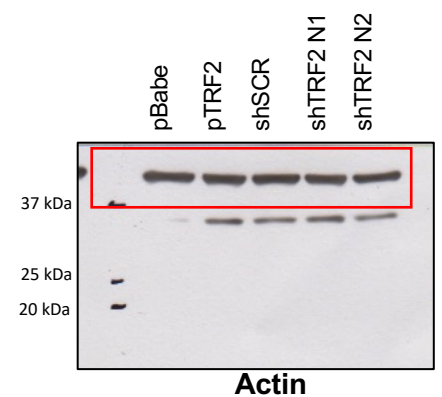

**F**

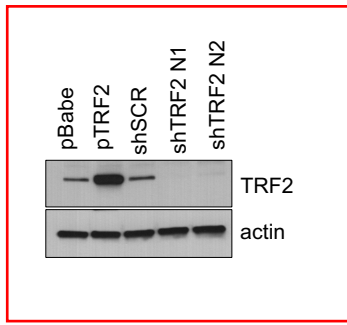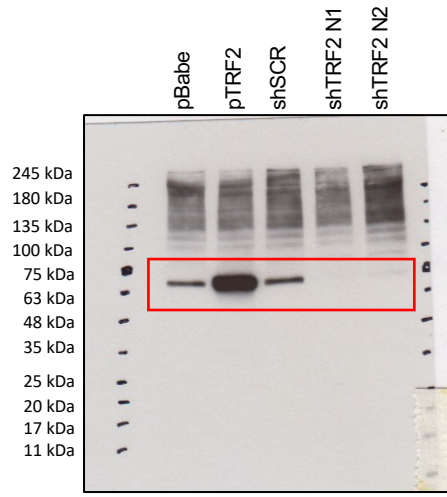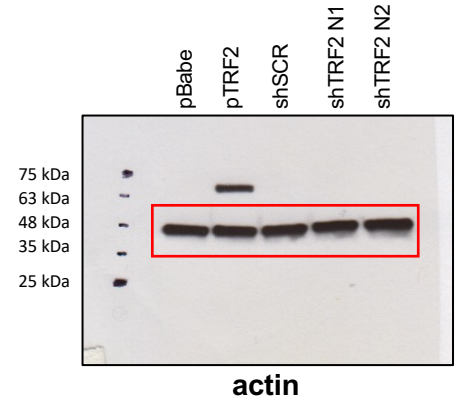

**TRF2**

**actin**

**G**

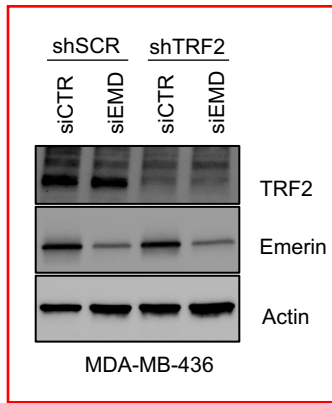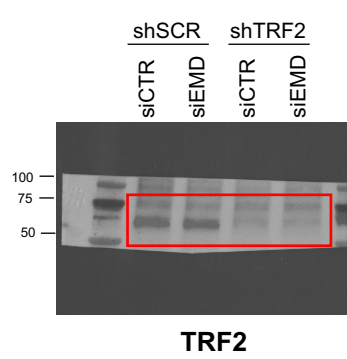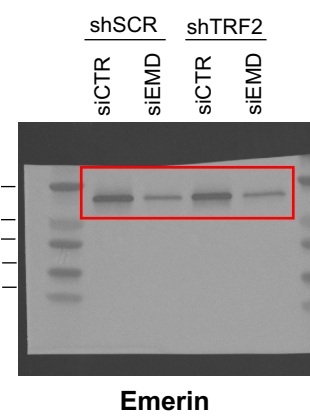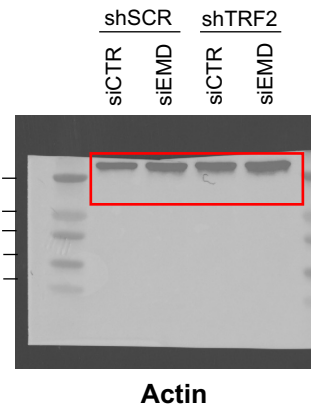

**TRF2**

**Emerin**

**Actin**

**I**

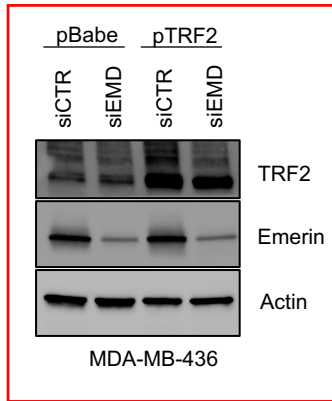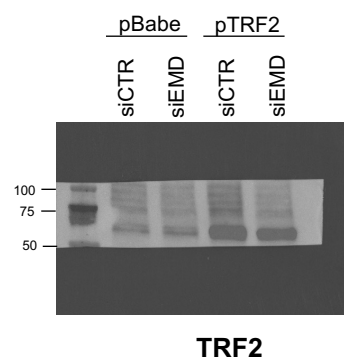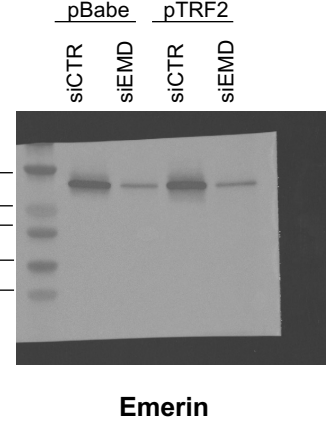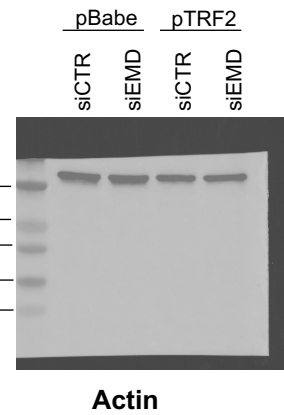

**TRF2**

**Emerin**

**Actin**

**J**

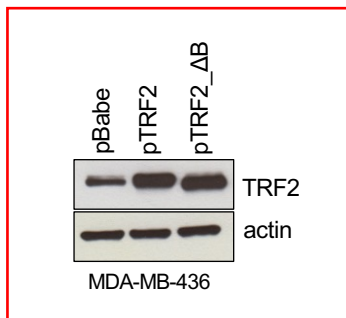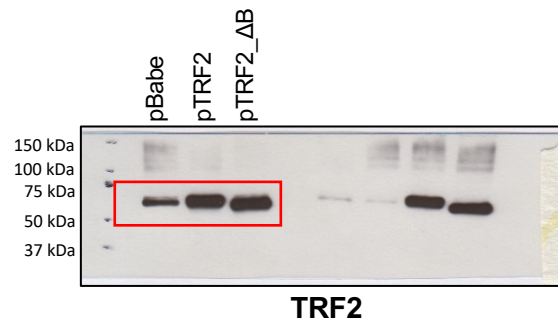

**TRF2**

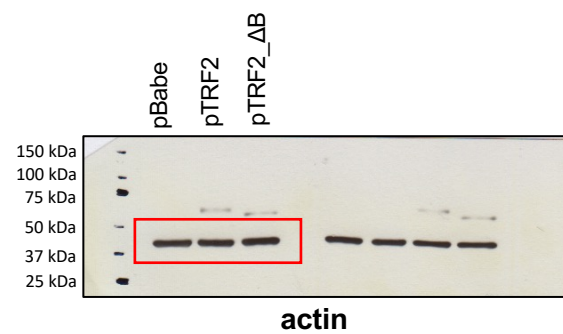

**actin**

C

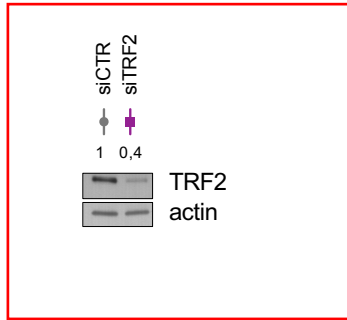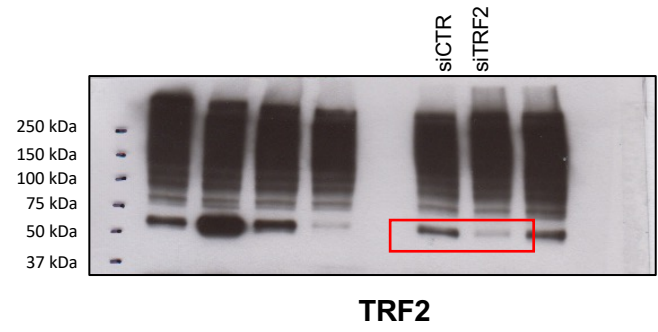

D

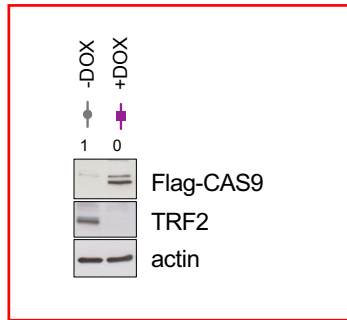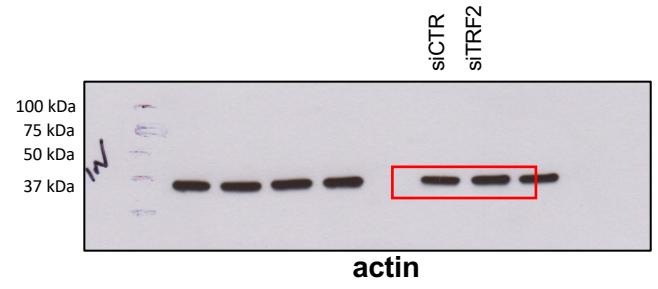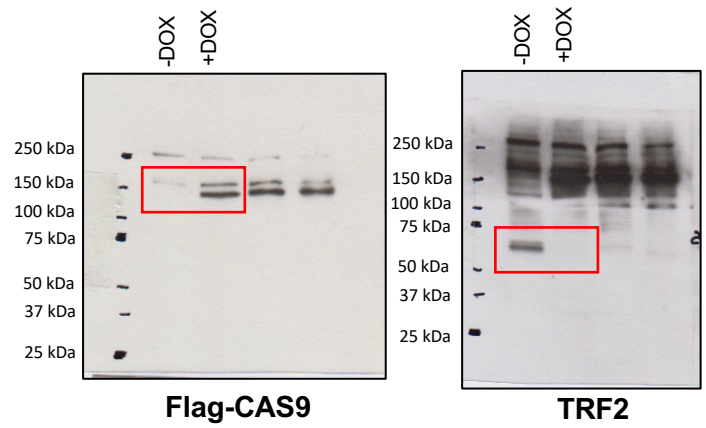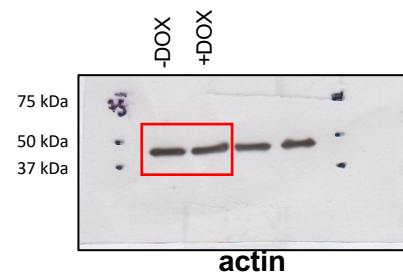

A

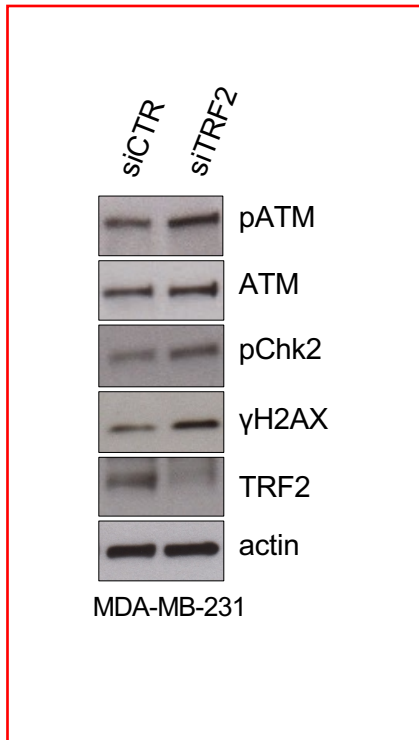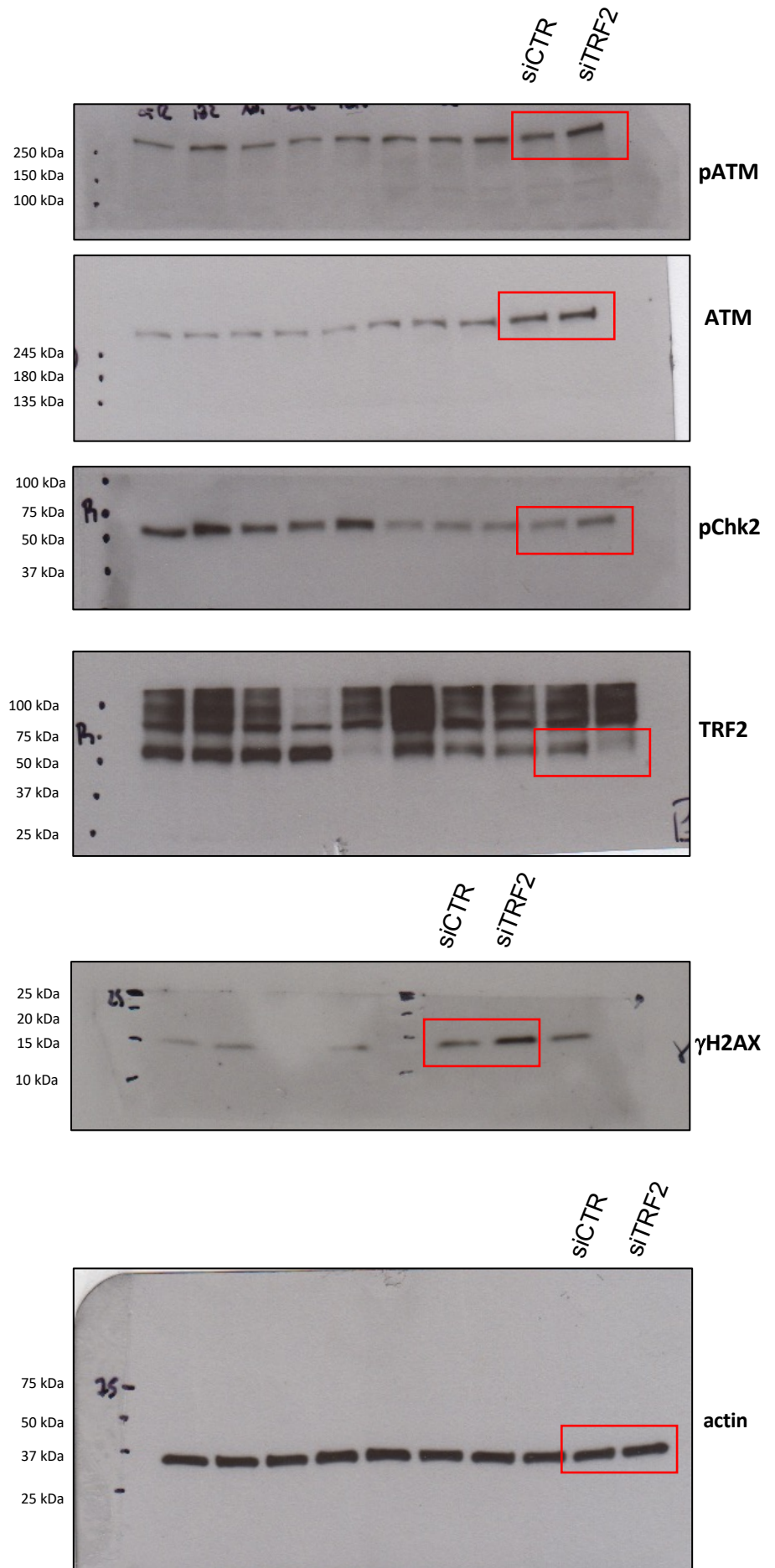

B

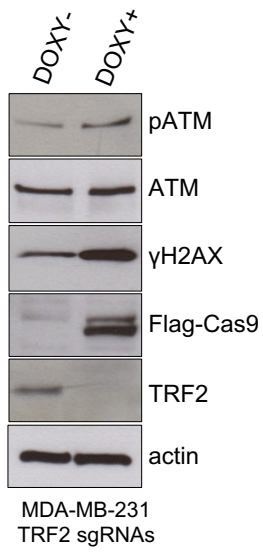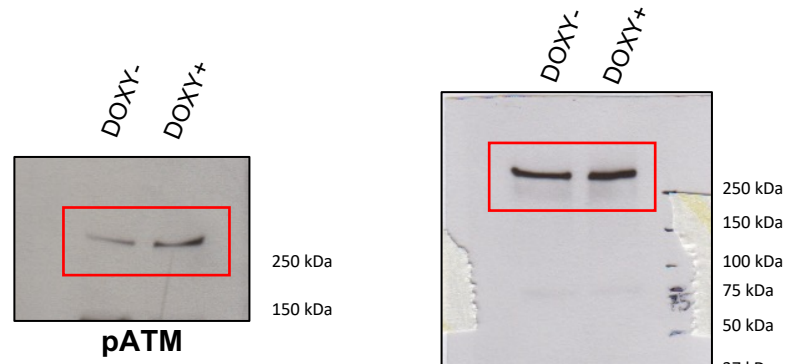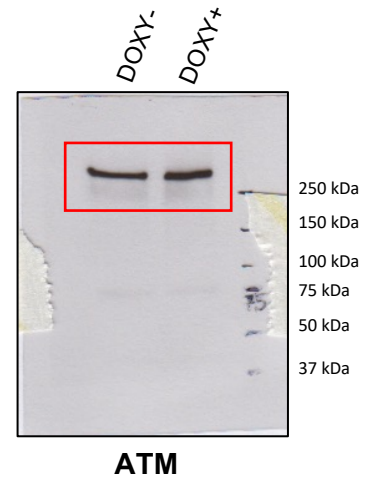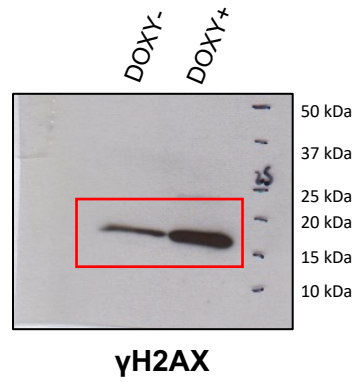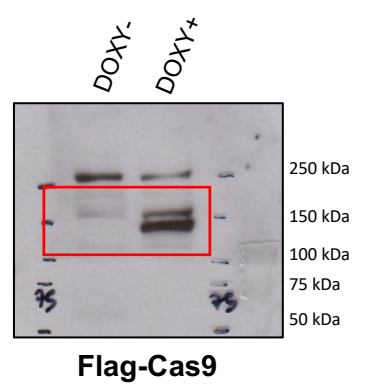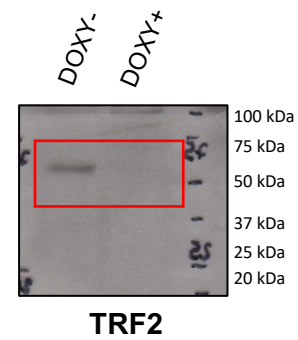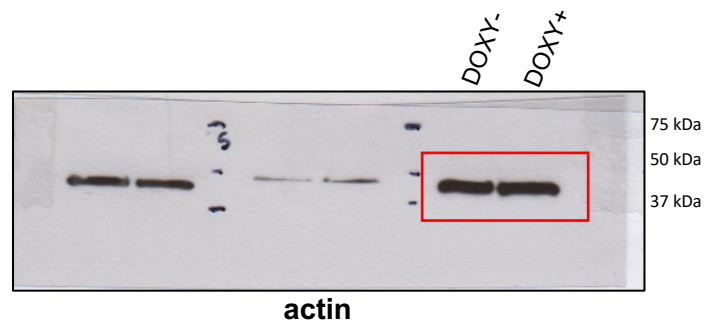

E

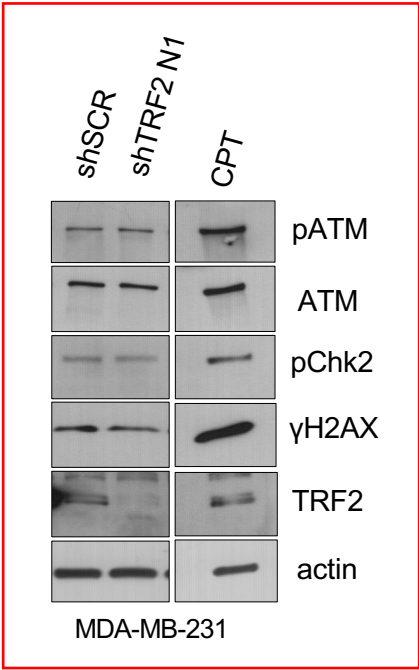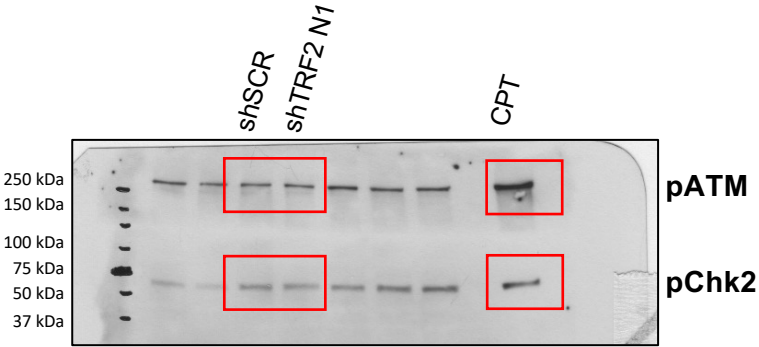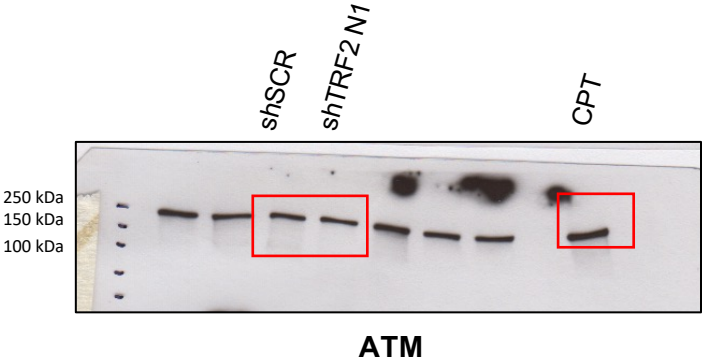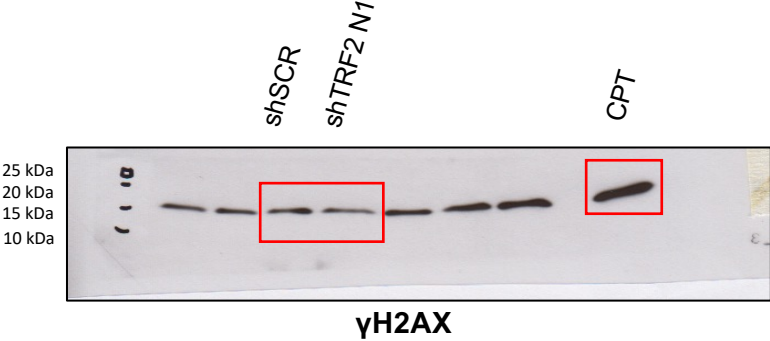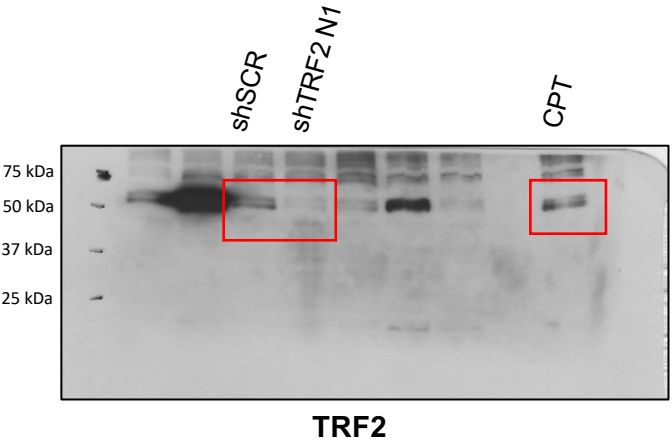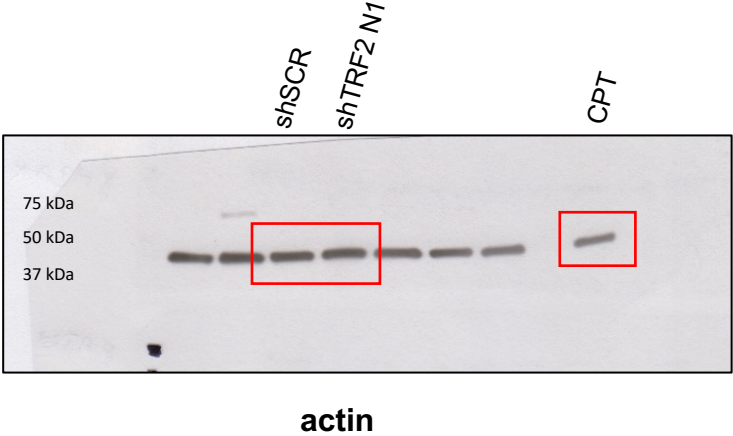

Supplement: Supplementary file 2 — Original Data File [file 41419_2025_7415_MOESM2_ESM.pdf]
